# Molecular basis of EphA2 recognition by gHgL from gammaherpesviruses

**DOI:** 10.1038/s41467-020-19617-9

**Published:** 2020-11-24

**Authors:** Chao Su, Lili Wu, Yan Chai, Jianxun Qi, Shuguang Tan, George F. Gao, Hao Song, Jinghua Yan

**Affiliations:** 1 College of Veterinary Medicine, China Agricultural University, Beijing, 100193 China; 2 CAS Key Laboratory of Microbial Physiological and Metabolic Engineering, Institute of Microbiology, Chinese Academy of Sciences, Beijing, 100101 China; 3 University of Chinese Academy of Sciences, Beijing, 100049 China; 4 CAS Key Laboratory of Pathogenic Microbiology and Immunology, Institute of Microbiology, Chinese Academy of Sciences, Beijing, 100101 China; 5 Research Network of Immunity and Health (RNIH), Beijing Institutes of Life Science, Chinese Academy of Sciences, Beijing, 100101 China

**Keywords:** Herpes virus, Virus-host interactions, X-ray crystallography

## Abstract

The human γ-herpesviruses Kaposi sarcoma associated herpesvirus (KSHV) and Epstein-Barr virus (EBV) are associated with many human malignancies. Viral glycoprotein H (gH) and glycoprotein L (gL) are crucial for the cell tropism by binding to specific receptors. Recently, EphA2 was identified as the specific entry receptor for both KSHV and EBV. Here, we characterized the crystal structures of KSHV gHgL or EBV gHgL in complex with the ligand binding domain (LBD) of EphA2. Both KSHV and EBV gHgL bind to the channel and peripheral regions of LBD primarily using gL. Extensive interactions with more contacts contribute to the higher affinity of KSHV gHgL to LBD than that of EBV gHgL. These binding characteristics were verified using cell-based fusion assays with mutations in key EphA2 residues. Our experiments suggest that multiple animal γ-herpesviruses could use EphA2 as an entry receptor, implying a potential threat to human health.

## Abstract (web summary)

EphA2 is the specific entry receptor for both human γ-herpesviruses Kaposi sarcoma associated herpesvirus (KSHV) and Epstein-Barr virus (EBV). Here, the authors present the crystal structures of the EphA2 ligand binding domain (LBD) bound to the viral glycoprotein gHgL from EBV and KSHV and further analyse EphA2 gHgL interactions with mutagenesis experiments in cell-based fusion assays and suggest that other animal γ-herpesviruses could also use EphA2 as an entry receptor.

## Introduction

Herpesviruses, enveloped double-stranded DNA viruses, have a broad host range. More than 100 species of herpesviruses are classified into the α-, β-, and γ-herpesviruses (https://talk.ictvonline.org/taxonomy/). The γ-herpesvirus comprises four genera: *Macavirus*, *Percavirus*, *Lymphocryptovirus*, and *Rhadinovirus*^1^. Epstein–Barr virus (EBV, *Lymphocryptovirus*) and Kaposi sarcoma-associated herpesvirus (KSHV, *Rhadinovirus*) are the only two human-infecting γ-herpesviruses. EBV and KSHV have many commonalities, such as an association with malignancies, broad cell tropism, and a similar entry process into host cells^2–4^. EBV primarily infects B cells and epithelial cells^5^, whereas KSHV predominantly infects B cells and endothelial cells.

The process by which herpesviruses enter cells is divided into three steps: attachment, receptor binding, and fusion. These steps are mediated by different viral glycoproteins^2,6^. Cell tropism is mainly determined by the interaction between a specific host receptor and the gHgL complex, with or without other glycoproteins^7^. For γ-herpesvirus, EBV gHgL forms a stable heterotrimeric complex with glycoprotein 42 (gp42). This complex, gHgL–gp42, binds to the B-cell-specific receptor human leukocyte antigen class II (HLA-II) protein to enter B cells^8^. The crystal structures of gHgL, gp42–HLA-II, and gHgL–gp42, in conjunction with the negative-stain electron micrographs of the structure of gHgL–gp42–HLA-II, provided evidence for the dynamic changes leading to the binding of gHgL–gp42 to HLA-II^9–12^. However, the receptor for EBV entry into the epithelial cells remained unknown until recently, when the ephrin receptor tyrosine kinase A2 (EphA2) was shown to bind to gHgL independent of gp42^13,14^. Similar to EBV gHgL, KSHV gHgL also binds to EphA2, to enter endothelial cells^15,16^. This interaction triggers the phosphorylation of EphA2 and subsequently induces the endocytosis of KSHV^15,16^. Other members of the EphA family, including EphA4, EphA5, and EphA7, also act as receptors for KSHV, allowing KSHV (but not EBV) to enter host cells (e.g., B cells)^17–19^.

EphA2 is an ephrin receptor tyrosine kinase. The ectodomain of this protein comprises the ligand-binding domain (LBD), the cysteine-rich domain, and the fibronectin III type repeats^20^. Via the LBD, EphA2 binds to its ephrin-A family ligands, such as ephrin-A1, ephrin-A2, and ephrin-A5, on the surfaces of adjacent cells^21–23^. Activation of EphA2 by these ligands regulates cellular properties associated with various cancers, including the actin cytoskeleton, cell–substrate adhesion, cell shape, and cell movement^24,25^. However, it remains unclear how KSHV and EBV use EphA2 for entry.

Here we report the crystal structures of EphA2 LBD bound to KSHV gHgL and EphA2 LBD bound to EBV gHgL. The overall structure of KSHV gHgL is similar to that of EBV gHgL. Both KSHV gHgL and EBV gHgL bind the channel and peripheral regions of LBD of EphA2 mainly via gL. Interactions between KSHV gHgL and EphA2 LBD are more extensive, with more contacts, as compared with those between EBV gHgL and EphA2 LBD, explaining the higher affinity for KSHV gHgL. These binding characteristics were further verified with cell-based fusion assays, using EphA2 mutated at key residues. The use of EphA2 as a high- and low-affinity receptor for KSHV and EBV, respectively, extends the cell tropism of these viruses in distinct modes. Of note, further cell-based fusion assays show that other γ-herpesviruses, from four different genera, also used human EphA2 as the entry receptor, implying that these γ-herpesviruses have the potential to infect humans. Our findings will help to drive the development of antiviral inhibitors against γ-herpesviruses.

## Results

### EBV gHgL binds to EphA2 LBD with a much lower affinity than KSHV gHgL

**Fig. 1:**
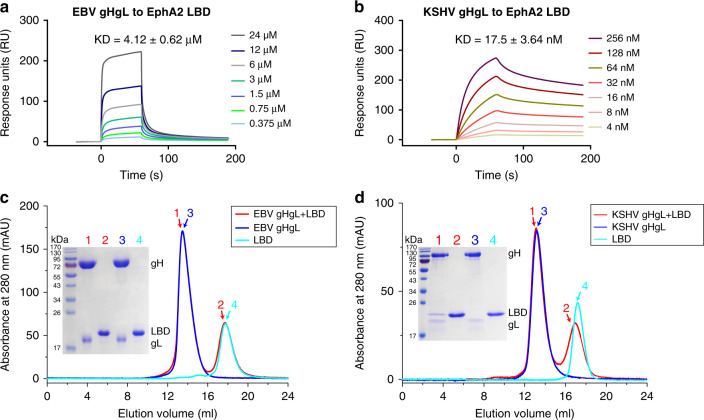
EBV gHgL binds to the LBD of EphA2 with much lower affinity than KSHV gHgL. **a**, **b** The binding affinity of EBV gHgL to LBD (**a**) and KSHV gHgL to LBD (**b**), as measured using SPR. EphA2 LBD was immobilized to chip CM5 and the binding affinities of various concentrations of EBV gHgL or KSHV gHgL were tested. The kinetic profiles are shown. KD values shown are the mean ± SEM of three independent experiments. The binding profiles were plotted by GraphPad Prism 8.0. **c** Analytical gel-filtration analyses of EBV gHgL–LBD (red), EBV gHgL (blue), and LBD (cyan) as measured using calibrated Superdex^®^ 200 Increase 10/300 GL columns (GE Healthcare). The chromatographs and SDS-PAGE profiles for each pooled sample (peaks 1–4) are shown. The SDS-PAGE results showed peak 1 contains only EBV gHgL proteins, indicating EBV gHgL and LBD proteins did not form a complex in the gel-filtration assays. **d** Analytical gel-filtration analyses of KSHV gHgL–LBD (red), KSHV gHgL (blue), and LBD (cyan) as measured using calibrated Superdex^®^ 200 Increase 10/300 GL columns (GE Healthcare). The SDS-PAGE results showed peak 1 contains both KSHV gHgL and LBD proteins, indicating these two proteins could form a complex in the gel-filtration assays. The gel-filtration chromatographs were plotted by OriginPro 8.5. Experiments have been repeated twice and similar results were observed. Source data are provided as a Source Data file.

Recent reports demonstrating that EBV and KSHV gHgLs share the same functional receptor EphA2^13,14^ prompted us to measure the affinity constants mediating these interactions via surface plasmon resonance (SPR) assays. Surprisingly, the LBD of EphA2 bound to EBV gHgL with ~4.12 μM affinity, which was ~230-fold lower than the affinity with which the LBD bound to KSHV gHgL (~17.5 nM affinity, Fig. 1a, b). We used gel-filtration chromatography assays to verify the observed binding affinities between EphA2 LBD and EBV gHgL or KSHV gHgL. As expected, EBV gHgL proteins did not form a complex with the LBD protein in the gel-filtration assays, whereas the KSHV gHgL protein did (Fig. 1c, d).

### Complex structure of KSHV or EBV gHgL bound to EphA2 LBD

**Fig. 2:**
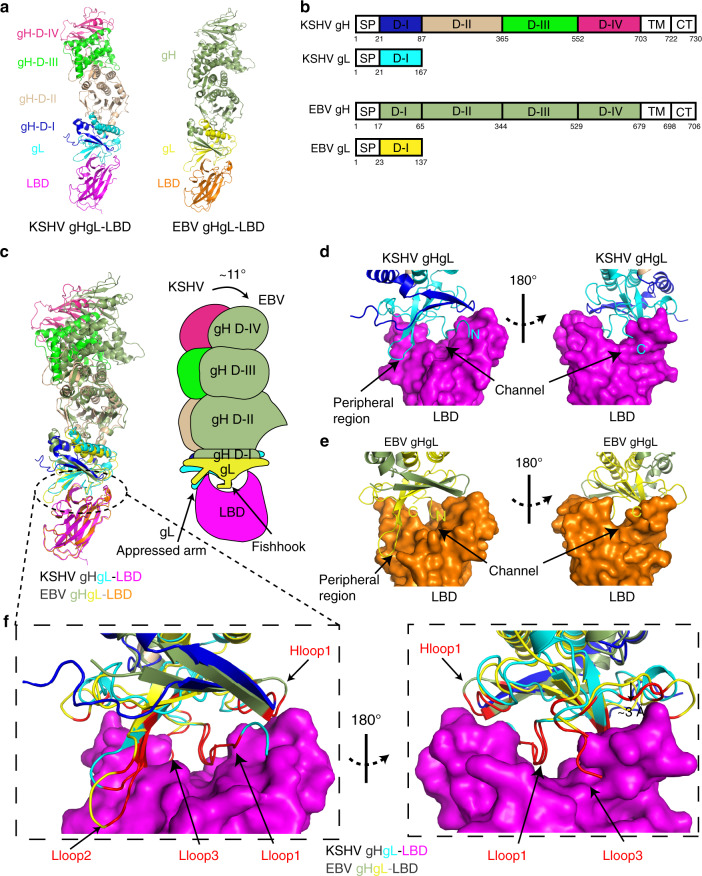
The overall structures of KSHV gHgL–LBD and EBV gHgL–LBD. **a** Cartoon structural representations of KSHV gHgL–LBD and EBV gHgL–LBD. KSHV gH is colored blue in D-I, wheat in D-II, green in D-III, and hotpink in D-IV. EBV gH is colored smudge. KSHV and EBV gL proteins are colored cyan and yellow, respectively. The LBDs of KSHV gHgL–LBD and EBV gHgL–LBD are colored magenta and orange, respectively. **b** Schematic representation of the KSHV gHgL and EBV gHgL. CT: C-terminal cytoplasmic tail domain, SP: signal peptide, TM: transmembrane. Different colored regions of KSHV gHgL correspond to the different structural domains shown in Fig. 2a. **c** Superimposition of KSHV gHgL–LBD and EBV gHgL–LBD. KSHV gHgL–LBD and EBV gHgL–LBD are colored as in Fig. 2a. Cartoon diagrams and binding mode pattern are shown. The shift angle and two binding regions of gL to LBD are shown. gL binds to LBD like a fishhook and appressed arm. **d**, **e** Binding mode of KSHV gHgL (**d**) and EBV gHgL (**e**) to LBD. The surface of LBD is shown, and the channel and peripheral regions of LBD are labeled. The N and C termini of gL are labeled N and C, respectively. **f** Comparison of the LBD-binding interfaces between KSHV gHgL and EBV gHgL. KSHV gHgL–LBD and EBV gHgL–LBD are colored as in Fig. 2a. The surface of LBD is shown. The LBD-binding residues of KSHV gHgL and EBV gHgL are colored red. The Hloop1, Lloop1–3, and β2 regions of KSHV gHgL are displayed.

To better understand the entry mechanism, we obtained the complex structure of KSHV gHgL bound to EphA2 LBD at a resolution of 3.2 Å and the complex structure of EBV gHgL bound to EphA2 LBD at a resolution of 3.0 Å (Supplementary Table 1). Overall, the structures of these two complexes were similar: both were shaped like elongated rods, about 150 Å in length and 30–55 Å in width (Fig. 2a). The structure of KSHV gHgL has not previously been reported. Based on the structural characteristics of EBV gHgL, the structure of KSHV gH was divided into four domains from the N terminus to the C terminus: D-I (A22-I87), D-II (R88-N365), D-III (H366-I552), and D-IV (P553-A703) (Fig. 2a, b). The KSHV gL protein mainly bound tightly to the D-I of the gH protein. We observed that both EBV and KSHV shared conserved disulfide bonds: five in gH and two in gL (Supplementary Fig. 1a, b).

Comparisons between KSHV gHgL and EBV gHgL indicate that the overall folds of gHgL is similar between the two structures, with a root-mean-square deviation (RMSD) of 4.12 Å (for 627 Cα atoms). Of note, gH D-I and gL shifted the most substantially, indicating that the binding interface between gH D-I and gL is flexible (Supplementary Fig. 1c). We then compared gL, gH, and each domain of gH between KSHV and EBV, respectively (Supplementary Fig. 1d, e). The amino acid sequence of the KSHV gL protein is 30 residues longer than that of EBV gL (Supplementary Figs. 2a and 3) and the RMSD of gL is 0.95 Å (for 75 Cα atoms). The Lloop2 of KSHV gL is shorter in length than that of EBV gL and shifted about 20° relatively to gL β1 and β2 (Supplementary Figs. 1d and 2a). The RMSD between KSHV and EBV for gH is 3.7 Å (for 526 Cα atoms), whereas the RMSDs between KSHV and EBV for the gH domains D-I, D-II, D-III, and D-IV are 2.86 Å (for 41 Cα atoms), 2.08 Å (for 168 Cα atoms), 1.75 Å (for 104 Cα atoms), and 0.92 Å (for 97 Cα atoms), respectively (Supplementary Fig. 1e). This indicates that the structure of the gH domain was more conserved closer to the transmembrane region.

### Structural comparison between KSHV gHgL bound to EphA2 LBD and EBV gHgL bound to EphA2 LBD

We compared the structures of the two complexes: KSHV gHgL bound to EphA2 LBD and EBV gHgL bound to EphA2 LBD. We found that, in both KSHV and EBV, LBD bound to the tip of gH D-I and to gL. The two LBD structures are similar, with only 0.62 Å RMSD (for 144 Cα atoms) between them (Supplementary Fig. 4a). Interestingly, KSHV gHgL and EBV gHgL were not arranged in a line, but exhibited an angle shift of about 11° relatively to the LBD (Fig. 2c). It suggested that interdomain arrangements differed between KSHV gHgL and EBV gHgL. KSHV gHgL and EBV gHgL mainly bound to two regions of the LBD. In both cases, the N terminus of gL inserts into the LBD channel and is shaped like a fishhook. The LBD channel was formed by the D-E, J-K loops, and G strand on the two sides, with the base made up of the M stand. In addition, Lloop2 and the β2 sheet of gL interact with the peripheral region (including the AC loop and D strand) of the LBD channel like an appressed arm (Fig. 2d, e). However, the binding details are distinct between KSHV and EBV. First, in KSHV, one more residue in the N-terminal region of Lloop1, which is hooked by residue R103 of LBD (Supplementary Fig. 4b), interacts with the LBD, as compared with EBV gL. Second, the C-terminal region of Lloop1 of KSHV gL is about 3 Å closer to LBD than that of EBV. Thus, more KSHV gL residues are involved in the interaction with the LBD (Fig. 2f). There are less contacts when KSHV gHgL bound to LBD through Lloop2 as compared with EBV, because the KSHV Lloop2 was shorter (Fig. 2f and Supplementary Table 2). The C terminus (Lloop3) of KSHV gL, which is involved in the interaction with LBD, has a longer sequence than that of EBV gL, and thus may be responsible for the increased association of KSHV gHgL with LBD (Fig. 2d–f).

### Binding interfaces between EphA2 LBD and KSHV gHgL or EBV gHgL

**Fig. 3:**
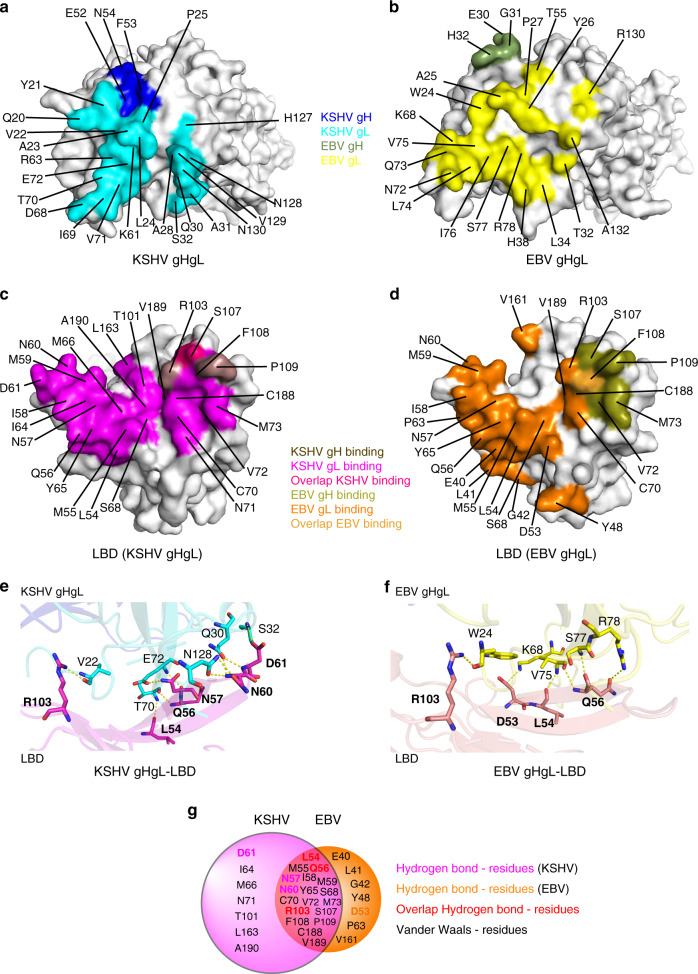
Detailed interactions between EBV or KSHV gHgL and EphA2 LBD. **a**, **b** Contact residues of the binding interface in the KSHV gHgL protein (**a**) or the EBV gHgL protein (**b**). The LBD-binding residues in KSHV gH and gL are colored blue and cyan, respectively; those in EBV gH and gL are colored smudge and yellow, respectively. **c**, **d** Contact residues of the binding interface in the LBD proteins. KSHV gH- and gL-binding residues are colored in violet and magenta, respectively; overlap binding residues are colored in red (**c**). EBV gH- and gL-binding residues are colored deep olive and orange, respectively; overlaping binding residues are colored yellow-orange (**d**). **e**, **f** Detailed hydrogen bond interactions between KSHV gHgL and LBD (**e**), and between EBV gHgL and LBD (**f**). **g** Detailed residues in LBD that interact with KSHV gHgL and EBV gHgL are shown in different colors; the area of each circle represents the number of contacts between gHgL and LBD. The residues binding to both KSHV and EBV gHgL with hydrogen bonds are colored in red. The residues binding only to KSHV gHgL with hydrogen bonds are colored magenta. The residues binding only to EBV gHgL with hydrogen bonds are colored orange.

We then analyzed the details of the bindings at the binding interfaces between KSHV gHgL and LBD, and between EBV gHgL and LBD. The buried surface was 1180 Å^2^ between KSHV gHgL and LBD, slightly larger than that of EBV (993.7 Å^2^). We found that 24 and 21 amino acids of KSHV gHgL and EBV gHgL, respectively, were involved in binding to LBD, with a distance cutoff of 4.5 Å (Supplementary Table 2). More than half of these amino acids were hydrophilic amino acids (charged or polar), located in Hloop1, the C terminus of Lloop1, Lloop2, and Lloop3. This indicates that these regions bind to LBD mainly through polar contacts. However, most of the residues in the N-terminal region of Lloop1 are hydrophobic (Fig. 3a, b and Supplementary Fig. 3), implying that both KSHV gHgL and EBV gHgL bind to the LBD channel primarily using Van der Waals forces. The KSHV gL residues involved in the binding to LBD are predominantly distributed in four regions: the N-terminal region of Lloop1, the C-terminal region of Lloop1, the Lloop2 and β2 region, and the Lloop3 region. In contrast, EBV gL residues are predominantly distributed in two regions: the N-terminal region of Lloop1, and the Lloop2 and β2 region (Fig. 3a, b and Supplementary Fig. 3). Notably, the KSHV gL residues (V22, Q30, S32, T70, E72, and N128) that form hydrogen bonds with LBD are widely distributed across the four regions, whereas the EBV gL residues (W24, K68, V75, S77, and R78) that form hydrogen bonds with LBD are only located in two regions (Fig. 3e, f and Supplementary Fig. 3). In addition, residues in the Hloop1 regions of both KSHV gH and EBV gH interact with LBD using Van der Waals (Fig. 3a, b).

For both KSHV gHgL and EBV gHgL, 25 amino acids in LBD are responsible for interaction by Van der Waals contacts; 18 of these residues are identical (Fig. 3c, d, g and Supplementary Table 2). However, there are a total of 317 contacts between KSHV gHgL and LBD, many more than total between EBV gHgL and LBD (177 contacts) (Supplementary Table 2). Furthermore, KSHV gHgL forms ten hydrogen bond interactions with LBD, compared with only six hydrogen bond interactions between EBV gHgL and LBD (Fig. 3e, f and Supplementary Fig. 5). LBD residues L54, Q56, and R103 are the key sites for the formation of hydrogen bond interactions. These sites are also conserved across with KSHV gHgL and EBV gHgL (Fig. 3g). Residues L54 and Q56 bind to the Lloop2 and β2 regions of both KSHV gL and EBV gL, whereas residue R103, which is conserved across EphA2, EphA4, EphA5, and EphA7, help to bind the residues in the N termini of KSHV gL and EBV gL (Fig. 3e, f and Supplementary Fig. 6a). A previous mutagenesis study demonstrated that LBD R103 contributes to the interactions between EphA2 and KSHV gHgL, as well as those between EphA2 and the ligands of EphA2^21,23,26^. Moreover, three additional LBD residues (N57, N60, and D61) form specific hydrogen bond interactions with KSHV gHgL, whereas only one additional LBD residue (D53) forms a hydrogen bond interaction with EBV gHgL (Fig. 3e, f). Thus, there are many additional binding contacts between KSHV gHgL and LBD as compared with that between EBV gHgL and LBD, especially the hydrogen bond interactions, results in the higher binding affinity between LBD and KSHV gHgL.

### Mutagenesis of the key interacting residues

**Fig. 4:**
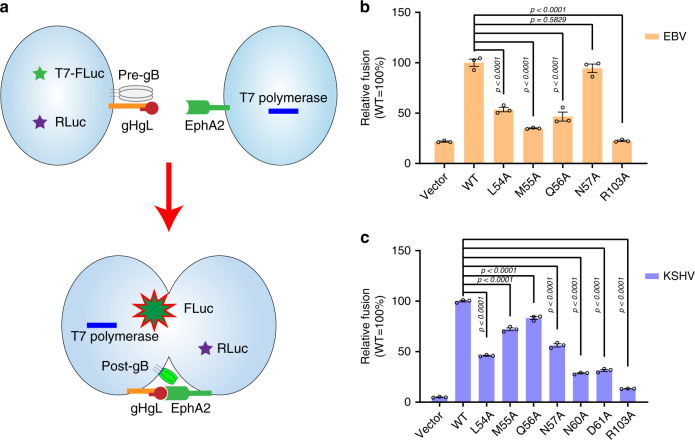
Key residues in the gHgL-binding site of EphA2 are critical for cell fusion. **a** Schematic diagram of the cell-based fusion assay. The cells expressing gB, gH, gL, and firefly luciferase reporter gene under the control of the T7 promoter were co-cultured with other cells expressing T7 polymerase and EphA2 proteins. Receptor EphA2 bound to gHgL, increasing the fusion of the two types of cells. T7 polymerase initiated the expression of firefly luciferase, which was detected using the Dual-Luciferase^®^ Reporter Assay System. *Renilla* luciferase was used as a transfection control. Pre-gB indicates the pre-fusion state of gB, whereas post-gB indicates the post-fusion state of gB. **b**, **c** Cell-based EBV (**b**) and KSHV (**c**) fusion assays were performed by co-culturing of HEK-293T cells transfected with plasmids expressing EphA2 and mutants, and HEK-293T cells transfected with plasmids expressing gB and EBV gHgL (**b**) or KSHV gHgL (**c**). Representative results from three experiments are shown. Relative fusion was normalized to that of wild type EphA2. The data are presented as mean ± SEM (*n* = 3 independent replicates). Statistical significance was analyzed using Ordinary one-way ANOVA with Dunnett’s multiple comparison test for multiple groups. The GenBank accession codes for gH, gL, and gB are shown in the Supplementary Table 3. Source data are provided as a Source Data file.

To investigate the roles of the residues important for the binding between LBD and gHgL described above, we introduced mutations in EphA2 and used virus-free cell-based fusion experiments to test the fusion efficiency (Fig. 4a). Based on our structural results, residues L54, M55, Q56, and R103 were chosen as key interacting residues for both KSHV gHgL and EBV gHgL; residues N57, N60, and D61 were also selected, as these played an important role in the interaction between LBD and KSHV gHgL. The cell-based fusion assays show that single alanine (A) substitutions at L54, M55, Q56, and R103 significantly reduce cell fusion of both KSHV gHgL and EBV gHgL. This reduction was especially noticeable at R103A, indicating that the hydrogen bond interaction formed between R103 and KSHV gL V22 or EBV gL W24 is crucial for the binding of EphA2 to KSHV gHgL or EBV gHgL (Fig. 4b, c). Notably, the N57A mutation only slightly disrupted the cell fusion of EBV, but significantly reduced that of KSHV (Fig. 4b, c). In addition, we found that single mutation at N60 and D61 dramatically affected the cell fusion of KSHV (Fig. 4c). These mutagenesis results are consistent with the structural analysis.

### Competitive binding with the EphA2 ligand ephrin-A1 and a neutralizing antibody (E1D1)

**Fig. 5:**
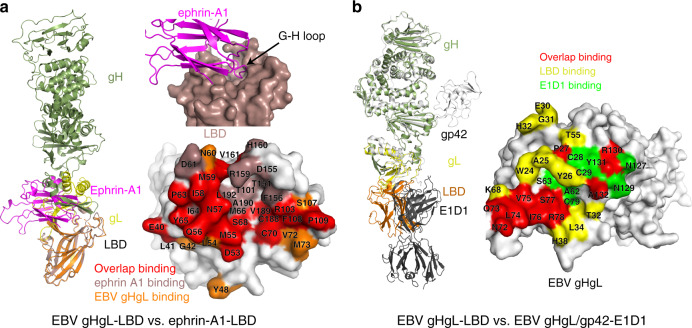
Structural comparisons of EBV gHgL–LBD to ephrin-A1–LBD and to EBV gHgL–gp42–E1D1. **a** Superimposition of the EBV gHgL–LBD with the ephrin-A1–LBD (PDB: 3HEI). The interface where ephrin-A1^21^ inserts into the LBD channel is shown. Details of the contact residues at the LBD-binding interface are shown. Ephrin-A1-binding residues are colored violet, EBV gHgL-binding residues are colored orange, and overlapping binding residues are colored red. **b** Superimposition of EBV gHgL–LBD and EBV gHgL–gp42–E1D1 (PDB: 5T1D). EBV gHgL–gp42–E1D1^12^ is colored gray. Details of the contact residues at the binding interface of EBV gHgL are shown. LBD-binding residues are colored yellow, E1D1-binding residues are colored green, and overlapping binding residues are colored red.

The native ligands of EphA2 are ephrin family proteins and include ephrin-A1, ephrin-A2, and ephrin-A5^21–23^. The crystal structure of the complex of EphA2 LBD and ephrin-A1 was previously reported^21^. In this structure, the G-H loop of ephrin-A1 inserts into the channel of LBD^21^. Structural comparisons between the ephrin-A1–LBD complex and the EBV gHgL–LBD complex show that the LBD-EBV gHgL and LBD-ephrin-A1-binding interfaces overlap. Over half of the residues responsible for ephrin-A1 binding are also used for gHgL–LBD binding (Fig. 5a). This result suggests that the binding of viral gHgL to EphA2 would preclude the binding of EphA2 to its ligand ephrin-A1.

It was previously reported that monoclonal antibody E1D1, which targets EBV gL, inhibited the entry of EBV into epithelial cells^12^. We compared the structure of the EBV gHgL–LBD complex with that of the EBV gHgL–gp42-E1D1 complex, and found that the interfaces partially overlap (Fig. 5b). Thus, the antibody E1D1 competitively bound to EBV gL, inhibiting the interaction between gHgL and its receptor EphA2.

### Structural differences among the gHgL proteins from α-, β-, and γ-herpesviruses

We compared the structure of KSHV gHgL to the structures of all other available gHgL proteins from α- and β-herpesviruses. The overall structure of both herpes simplex virus 2 (HSV-2) gHgL^27^ and varicella-zoster virus (VZV)^28^ gHgL resemble a boot (Supplementary Fig. 7a, b), whereas KSHV and human cytomegalovirus (HCMV)^29^ adopt an elongated rod-like configuration (Supplementary Fig. 7c). KSHV gH D-I and gL were much shorter than those of HCMV (Supplementary Fig. 7c) and the structures of gH D-I and gL differed substantially among KSHV, HSV-2, VZV, and HCMV, especially with respect to the arrangements of gL (Supplementary Fig. 7a–c). Sequence alignments also indicated that gH D-I and gL were more variable in KSHV as compared with other herpesviruses (Supplementary Fig. 8a, b). Thus, the binding modes of gH to gL are distinct among the α-, β-, and γ-herpesviruses. The differences among these arrangements may give rise to the diverse receptor tropism.

### EphA2 might serve as an entry receptor for multiple γ-herpesviruses

**Fig. 6:**
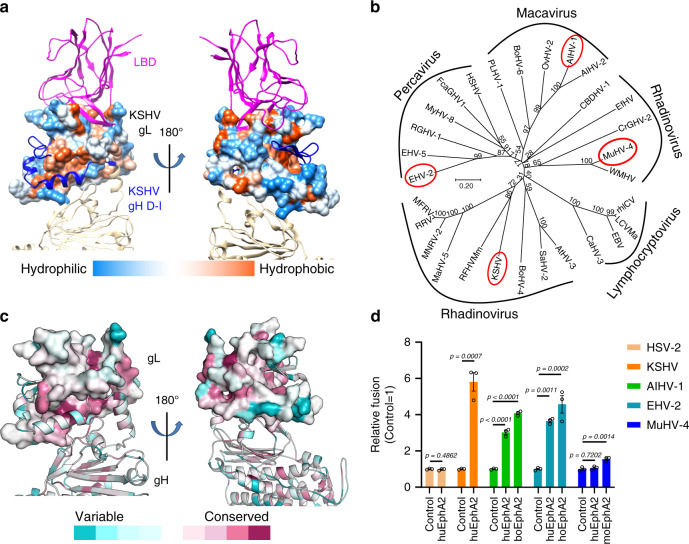
EphA2 may serve as an entry receptor for multiple γ-herpesviruses. **a** The structure of KSHV gL shows hydrophobic (orange) surfaces on both the EphA2 and the gH-binding faces. Molecular surfaces are colored according to hydrophobicity, with blue, white, and orange corresponding to the most hydrophilic, neutral, and hydrophobic patches, respectively. **b** Phylogenetic analysis of the gL proteins from 29 γ-herpesviruses, as generated by MEGA 10.1^46^. Analyses were performed using the neighbor-joining method. The viruses selected for the cell fusion experiments are shown in red ellipses. **c** Conserved gL sequences mapped onto the protein surface. The gL surface is gradiently colored by the ConSurf server^47^, based on degree of conservation as indicated by the alignment of gL sequences from 29 γ-herpesviruses: the most conserved regions are dark magenta and the most divergent regions are dark cyan. **d** Cell-based fusion assays were performed by co-culturing of HEK-293T cells transfected with EphA2 and HEK-293T cells transfected with plasmids expressing viral gHgL and gB. The *Macavirus* AIHV-1, *Percavirus* EHV-2, and *Rhadinovirus* MuHV-4 were randomly selected for testing. KSHV was used as the positive control and HSV-2 was used as the negative control. Representative results from three experiments are shown. Relative fusion was normalized to the empty vector. The data are presented as mean ± SEM (*n* = 3 independent replicates). Unpaired Student’s *t*-test was used for comparing two groups and ordinary one-way ANOVA with Dunnett’s multiple comparison test for multiple comparisons. boEphA2: bovine EphA2; hoEphA2: horse EphA2; huEphA2: human EphA2; moEphA2: mouse EphA2. The GenBank accession codes of gH, gL, and gB are shown in the Supplementary Table 3. Source data are provided as a Source Data file.

The similarities of EphA2-binding modes between the two γ-herpesviruses that infect humans prompted us to test whether other γ-herpesviruses also use EphA2 as a receptor. Sequence alignments of gH (Supplementary Fig. 9) and gL (Supplementary Fig. 3) from 29 γ-herpesviruses, in 4 genera (*Macavirus*, *Percavirus*, *Lymphocryptovirus*, and *Rhadinovirus*), indicate that most of γ-herpesviruses gH proteins and all of γ-herpesviruses gL proteins contain conserved cysteines that form disulfide bond interactions. Furthermore, we also found that the residues in the core secondary structures of KSHV and EBV gL were more conserved across the γ-herpesviruses than residues in other positions (Supplementary Fig. 3). Analyses of the structural hydrophobicity of KSHV gL identified hydrophobic regions on the binding surfaces of EphA2 and gH (Fig. 6a). The phylogeny of the gL proteins from the 29 γ-herpesviruses recovered the 4 genera in 5 clusters, with *Rhadinovirus* encompassing two clusters (Fig. 6b). The most conserved regions of gL are the hydrophobic gH-binding area and the cysteines located on the N-terminal loop. Although other EphA2-binding residues are variable, the amino acid characteristics are similar (Fig. 6c).

We then selected three viruses for further functional analysis: Alcelaphine gammaherpesvirus 1 (AIHV-1), Equid gammaherpesvirus 2 (EHV-2), and Murid gammaherpesvirus 4 (MuHV-4): each of these viruses was from one of the other three clusters in the gL (Fig. 6b). Plasmids expressing either gH or gL proteins from each of the three viruses were synthesized, respectively, and cell-based fusion assays were performed to determine fusion efficiency. As AIHV-1 infects cows, EHV-2 infects horses, and MuHV-4 infects mice, plasmids expressing bovine, equid, and murine EphA2 were also synthesized and evaluated. Cell fusion results showed that the gHgL of HSV-2, which is an α-herpesvirus, could not utilize human EphA2 to trigger cell fusion (Fig. 6d), whereas KSHV could, indicating that the assay was reliable. Importantly, the gHgL proteins from AIHV-1 and EHV-2 used bovine EphA2 and equid EphA2, respectively, to trigger cell fusion. As the gHgL-binding sites for EphA2 were conserved across species (Supplementary Fig. 6b, c), the gHgL proteins of AIHV-1 and EHV-2 could therefore also use human EphA2 to trigger cell fusion (Fig. 6d). Interestingly, MuHV-4 gHgL had the weak ability to use mouse EphA2, but not human EphA2, to trigger cell fusion. Indeed, MuHV-4 may mainly utilize other members of Eph family as the receptors, such as Rhesus monkey rhadinovirus (RRV)^30^. These results suggest that γ-herpesviruses may potentially bind to human EphA2 (or other members of Eph family), highlighting their potential threat to human health.

## Discussion

In this study, we investigated the structures of both KSHV gHgL and EBV gHgL bound to the LBD of EphA2. KSHV gHgL and EBV gHgL form similarly elongated rod shapes and bind to LBD primarily through two regions: the N terminus of gL inserted into the channel of LBD using Van der Waals forces, similar to ephrin-A1, whereas Lloop2 and β2 interact with the peripheral region of LBD through polar contacts. However, compared with the structure of EBV gHgL–LBD, the structure of KSHV gHgL–LBD includes a crooked elongated rod, which bends from gL to gH D-IV. Moreover, extensive interactions with more contacts contribute to the higher affinity of LBD to KSHV gHgL than that of EBV gHgL, which was supported by SPR experiments and gel-filtration chromatography experiments. Furthermore, our alignment of LBD sequences from EphA2, EphA4, EphA5, and EphA7 show that the EphA2 residues involved in the formation of hydrogen bonds with KSHV gHgL and EBV gHgL are not conserved across EphA proteins, with the exception of R103. Indeed, residue R103 is critical for the binding of LBD to KSHV gHgL and EBV gHgL (Supplementary Fig. 6a). Across the EphA proteins, there are only ten and eight conserved EphA2 residues involved in binding to KSHV gHgL and EBV gHgL, respectively, with Van der Waals forces (Supplementary Fig. 6a). Therefore, although the structure and the ligand-binding mode of EphA2 are similar to those of other members of the Eph family, including EphA4, EphB2, and EphB4^21^^,^^31–33^, EBV gHgL could hardly bind to the EphA4 after weakly binding EphA2^13^, whereas KSHV gHgL could weakly bind other members of EphA family such as EphA4, EphA5, and EphA7^16,18,19,30^, and was thus able to enter cells.

Interestingly, the properties of LBD-binding amino acids in KSHV gHgL and EBV gHgL were also observed in other γ-herpesviruses. Although no previous studies have reported that EphA2 acts as a receptor for other members of γ-herpesviruses, here we showed that three γ-herpesviruses, AIHV-1, EHV-2, and MuHV-4, bound to EphA2 with varying degrees of efficiency. As KSHV, EBV, AIHV-1, EHV-2, and MuHV-4 fall into four different γ-herpesvirus genera, the gHgL proteins of other γ-herpesviruses may potentially interact with EphA2 proteins. However, we cannot exclude the possibility that gHgL proteins from other γ-herpesviruses bind to other members of Eph family. For example, some γ-herpesviruses only utilize other members of the Eph family as receptors, such as RRV, whose gHgL binds to several Eph family proteins (e.g., EphA4, EphA7, EphB2, and EphB3), but not EphA2^30^. As EphA2 protein sequences are highly conserved across species, and as both AIHV-1 and EHV-2 gHgL proteins bind to human EphA2, these γ-herpesviruses have the cross-species infection potential.

Consistent with our results, previous studies demonstrated that EBV gHgL–gp42 binds to HLA-II with nanomolar affinity^11^, and that gHgL interacts with EphA2 with micromolar affinity^13^. KSHV gHgL and EBV gHgL bound to different receptors with high and low affinities, suggesting that KSHV and EBV may regulate the infection of various target cells based on their affinity to the host receptor. Based on our result, we defined HLA-II and EphA2 as the high- and low-affinity receptors for EBV, respectively. EphA4, EphA5, and EphA7 are low-affinity receptors for KSHV, in constrast to the high-affinity receptor EphA2. However, EBV and KSHV used high- and low-affinity receptors to determine cell tropism in different ways (Supplementary Fig. 10a, b).

The surfaces of EBV virions carry two different types of gHgL complexes: the heterodimeric gHgL complex and the heterotrimeric gHgL–gp42 complex^34^. EBV virions produced in B cells have small amounts of the gHgL–gp42 complex due to degradation of gp42 upon interaction with HLA-II in the endoplasmic reticulum^34^. In contrast, EBV virions produced in epithelial cells which lack the HLA-II protein, are rich in the gHgL–gp42 complex^34^. However, the gHgL–gp42 proteins on the surface of the gHgL–gp42-rich virions do not help the virions enter the epithelial cells, because soluble gp42 proteins inhibit EphA2-mediated epithelial cell fusion^13,35,36^. Even though gHgL–gp42 proteins may weekly interact with the low-affinity receptors EphA2 on the epithelial cell, this weak binding means that these virions are easily separated from the surface of epithelial cell, while the strong binding of the gHgL–gp42 proteins to high-affinity receptor HLA-II helps the virions to easily enter B cells (Supplementary Fig. 10a)^11,34^. Conversely, although the gHgL-rich virions infect B cells via the interactions between the small amounts of gHgL–gp42 and the high-affinity receptor HLA-II, the larger amounts of gHgL protein virion surfaces help the virions to bind to the low-affinity receptor EphA2 on the epithelial cell for entry. The gHgL–gp42 proteins may also help virions to attach to the surfaces of epithelial cells. Therefore, these virions tend to infect epithelial cells (Supplementary Fig. 10a)^34^. That is, the EBV virus infects epithelial cells and B cells alternately, not only due to the gp42 switch, but also with the help of the high- and low-affinity receptors. The high-affinity receptor HLA-II is only expressed in antigen-presenting cells, such as B cells, dendritic cells, and macrophages, whereas the low-affinity receptor EphA2 is widely expressed in epithelial cells, endothelial cells, and fibroblasts^18^. Therefore, high-affinity receptors ensure persistent EBV infection in HLA-II-positive B lymphocytes, whereas the emergence of low-affinity receptors greatly diversifies the types susceptible to EBV infection.

As a high-affinity receptor, EphA2 helps KSHV enter large numbers of epithelial cells, endothelial cells, and fibroblasts^18^. However, EphA2 is not expressed in B cells^18^, which are the main target cell of KSHV infection. Thus, KSHV must utilize other receptors. As EphA4, EphA5, and EphA7, which are low-affinity receptors for KSHV, are expressed in B cells, these receptors play a crucial role in the infection of B cells by KSHV^16,18,19,30^. Multiple types of low-affinity receptors together ensure that KSHV infects B cells efficiently (Supplementary Fig. 10b). In addition, KSHV utilizes specific receptors, bound to other viral glycoproteins, such as DC-SIGN and xCT/CD98, to broaden the types of cells infected^2^. The γ-herpesvirus RRV, whose gHgL sequence is more similar to that of KSHV gHgL than EBV gHgL (Supplementary Figs. 3 and 9), has a similar receptor-binding mode to that of KSHV. The high-affinity receptor for RRV gHgL is EphB3 but not EphA2, and RRV gHgL also weakly interacts with other members of the Eph family, including EphA4, EphA7, and EphB2^30^.

In conclusion, our work revealed the structural basis of EphA2 recognition by γ-herpesvirus gHgL. The existence of both high- and low-affinity receptors not only ensures a wide range of cell tropism for KSHV and EBV, but also maintains the persistent infection of these viruses in specific cells. Our results help to clarify the entry mechanisms of KSHV and EBV, and may inform the development of specific drugs to target the γ-herpesvirus entry process. However, how γ-herpesvirus gHgL transfers signals to glycoprotein B (gB) after binding specific receptors remains unclear; this question requires future study.

## Methods

### Gene cloning, expression, and protein purification

The DNA sequences encoding KSHV gH (residues 22–698, GenBank: YP_001129375.1), KSHV gL (residues 21–167, GenBank: YP_001129399.1, C-terminal 6× His-tag), and human EphA2-LBD (residues 28–206, GenBank: NP_004422.2,C-terminal 6× His-tag) were separately synthesized and cloned into the baculovirus transfect vector pFastBac1^37,38^, and EBV gH (residues 18–679, GenBank: YP_401700.1) and EBV gL (residues 24–137, GenBank: YP_401678.1, C-terminal 6× His-tag) were synthesized and cloned into pFastBac Dual vector^10,21^. A gp67 signal sequence was added at the N terminus of genes in both vectors. Transfection and virus amplification were conducted with Sf9 cells and the recombinant gHgL and LBD proteins were expressed in High Five cells. The culture supernatant containing the proteins was collected and then purified by affinity chromatography using the HisTrap HP 5 ml columns (GE Healthcare). The target protein was eluted with the elution buffer (20 mM Tris, 150 mM NaCl pH 8, and 300 mM imidazole). For KSHV gHgL, the fractions of KSHV gHgL proteins were pooled, dialyzed in 20 mM Tris, 50 mM NaCl pH 7.4, and applied to a ResourceTM S cation exchange chromatography equilibrated with the same buffer, and eluted with an increasing linear 50 mM to 1 M NaCl gradient in 20 mM Tris-HCl buffer pH 7.4. The fractions were then purified by HiLoad 16/600 Superdex® 200 pg column in buffer A containing 20 mM Tris, 150 mM NaCl pH 8.0. For EBV gHgL, the fractions were pooled, dialyzed in 20 mM Tris, 90 mM NaCl pH 8, and applied to a ResourceTM Q anion exchange chromatography equilibrated with the same buffer, and eluted with an increasing linear 90 mM to 1 M NaCl gradient in 20 mM Tris-HCl buffer pH 8.0. The fractions were further purified by HiLoad 16/600 Superdex® 200 pg column in buffer A. The LBD proteins expressed by insect cells were purified via His-tag affinity and further purified on HiLoad 16/600 Superdex® 75 pg column (GE Healthcare) in buffer A. We also cloned EphA2-LBD (residues 26–200, C-terminal 6× His-tag) into pET-21a (+) vector and the vector was transformed into *Escherichia coli* strain BL21 (DE3). The inclusion bodies of LBD protein were purified and then refolded in the refolding buffer (100 mM Tris-HCl pH 8.0, 2 mM EDTA, 10% glycerol, 400 mM l-arginine, 0.5 mM oxidized glutathione, and 5 mM reduced glutathione) by gradual dilution. The refolded protein was dialyzed into buffer A, followed by purified in buffer A by gel-filtration chromatography on HiLoad 16/600 Superdex® 75 pg column (GE Healthcare).

### Complex preparation and crystallization

Purified KSHV gHgL and LBD proteins were mixed and incubated on ice for 1 h. These mixtures were subsequently purified using Superdex® 200 Increase 10/300 GL columns (GE Healthcare) with 20 mM Tris pH 8.0, 50 mM NaCl. The KSHV gHgL–LBD complex was concentrated to ~7 mg/mL for crystallization. Purified EBV gHgL and LBD proteins were mixed in a 1:1.5 ratio at a concentration of 10 mg/mL and incubated on ice overnight for crystallization. All crystallizations were performed using the vapor-diffusion sitting-drop method, with 0.8 μL protein mixing with 0.8 μL reservoir solution at 18 °C or 4 °C. High-quality KSHV gHgL–LBD complex crystals were obtained using 0.2 M potassium phosphate dibasic and 20% w/v polyethylene glycol 3350; high-quality EBV gHgL–LBD complex crystals were obtained using 1% w/v tryptone, 0.001 M sodium azide, 0.05 M HEPES sodium pH 7.0, and 12% w/v polyethylene glycol 3350. The crystals of the KSHV gHgL–LBD complex were further optimized by seeding. Diffraction data were obtained using 0.25 M potassium phosphate dibasic and 17% w/v polyethylene glycol 3350 at a concentration of 6.2 mg/mL at 18 °C.

### Data collection and structure determination

To collect the diffraction data, all crystals were flash-cooled in liquid nitrogen after a short incubation in reservoir solution with the addition of 20% (v/v) glycerol. X-ray diffraction data of KSHV gHgL–LBD complex were collected at Shanghai Synchrotron Radiation Facility (SSRF) BL17U, whereas the data of EBV gHgL–LBD complex were collected at BL19U. The data were indexed, integrated, and scaled with HKL2000^39^. The structures of KSHV gHgL–LBD and EBV gHgL–LBD were determined by molecular replacement method using Phaser^40^ with the previously reported structures of EBV gHgL (PDB: 5W0K) and EphA2-LBD (PDB: 3FL7) as search models. The atomic models were built with Coot^41^ and the refinements were done with Phenix.refine^42^. The stereochemical quality of final models was assessed with MolProbity^43^. Data collection, processing, and refinement statistics are summarized in Supplementary Table 1. All structural figures were generated using PyMOL software (https://pymol.org/2/) and UCSF Chimera 1.14^44^.

### SPR assay

The SPR assay was performed using a BIAcore 8 K with a CM5 chip (GE Healthcare) at 25 °C. The buffer system was PBST (10 mM Na_2_HPO_4_, 2 mM KH_2_PO_4_ pH 7.4, 137 mM NaCl; 2.7 mM KCl, 0.005% Tween 20). The LBD protein was immobilized on the CM5 chip using standard amine coupling chemistry. We serially diluted KSHV gHgL (4, 8, 16, 32, 64, 128, and 256 nM) and EBV gHgL (0.375, 0.75, 1.5, 3, 6, 12, and 24 μM, respectively). The dilutions were separately flowed over the chip surface to measure the response units. The binding kinetics of KSHV gHgL–LBD and EBV gHgL–LBD were analyzed separately, using a 1:1 binding model, in Biacore Insight Evaluation, version 1.0.5.11069.

### Cell fusion assay

The cell-based fusion experiment was performed as previously reported^14^. The T7 polymerase and T7 firefly luciferase expression plasmids were constructed previously in our lab^45^. Effector HEK-293T cells and target HEK-293T cells were seeded and grown to ~70% confluence in six-well plates. Effector HEK-293T cells were transiently transfected with plasmids containing gH, gL, gB, T7 firefly luciferase, or *Renilla* luciferase (internal control, pRL-TK). Target HEK-293T cells were transiently transfected with T7 polymerase and pEGFP-N1-EphA2 (or empty vectors or mutants or from various species) for 24 h. After transfection, effector and target cells were digested and then co-cultured at a 1:1 ratio in 24-cell plates. After 24 h, the cells were washed with phosphate-buffered saline and lysed with lysis buffer. Next, 10 μl lysed cells and 50 μl luciferase assay reagent (Promega) were added to each well into 96-well plates to measure the firefly luciferase activity and *Renilla* luciferase activity using a GloMax-96 Microplate Luminometer. Data were analyzed using GraphPad Prism 8.0.

### Statistical analyses and reproducibility

Statistical analysis was conducted on data from three biologically independent experimental replicates. Error bars displayed on graphs represent the mean ± SEM of three independent experiments. Statistical significance was analyzed using unpaired Student’s *t*-test for two groups or Ordinary one-way anaysis of variance with Dunnett’s multiple comparison test for multiple groups. *p < 0.05, **p < 0.01, ***p < 0.001, and ****p < 0.0001 were considered significant. All analyses were performed using GraphPad Prism 8.0. Figures 1a, b, 4b, c, and 6d have been repeated three times and Fig. 2c–d have been repeated twice, and similar results were observed.

### Reporting summary

Further information on research design is available in the Nature Research Reporting Summary linked to this article.

## Supplementary information

Supplementary Information

Peer Review

Reporting Summary

## Data Availability

The data that support the findings of this study are available from the corresponding author upon reasonable request. The accession numbers for the atomic coordinates and diffraction data reported in this paper are PDB 7CZE (crystal structure of EphA2 LBD with EBV gHgL complex) and 7CZF (crystal structure of EphA2 LBD with KSHV gHgL complex). Source data are provided with this paper.

## Source data

Source Data

## References

[1] Davison AJ (2009). The order herpesvirales. Arch. Virol..

[2] Mohl BS, Chen J, Longnecker R (2019). Gammaherpesvirus entry and fusion: a tale how two human pathogenic viruses enter their host cells. Adv. Virus Res..

[3] Jha HC, Pei Y, Robertson ES (2016). Epstein-Barr virus: diseases linked to infection and transformation. Front. Microbiol..

[4] Li S, Bai L, Dong J, Sun R, Lan K (2017). Kaposi’s sarcoma-associated herpesvirus: epidemiology and molecular biology. Adv. Exp. Med. Biol..

[5] Thorley-Lawson DA, Hawkins JB, Tracy SI, Shapiro M (2013). The pathogenesis of Epstein-Barr virus persistent infection. Curr. Opin. Virol..

[6] Azab W, Osterrieder K (2017). Initial contact: the first steps in herpesvirus entry. Adv. Anat. Embryol. Cell Biol..

[7] Heldwein EE (2016). gH/gL supercomplexes at early stages of herpesvirus entry. Curr. Opin. Virol..

[8] Li Q (1997). Epstein-Barr virus uses HLA class II as a cofactor for infection of B lymphocytes. J. Virol..

[9] Mullen MM, Haan KM, Longnecker R, Jardetzky TS (2002). Structure of the Epstein-Barr virus gp42 protein bound to the MHC class II receptor HLA-DR1. Mol. Cell.

[10] Matsuura H, Kirschner AN, Longnecker R, Jardetzky TS (2010). Crystal structure of the Epstein-Barr virus (EBV) glycoprotein H/glycoprotein L (gH/gL) complex. Proc. Natl Acad. Sci. USA.

[11] Sathiyamoorthy K (2014). Assembly and architecture of the EBV B cell entry triggering complex. PLoS Pathog..

[12] Sathiyamoorthy K (2016). Structural basis for Epstein-Barr virus host cell tropism mediated by gp42 and gHgL entry glycoproteins. Nat. Commun..

[13] Chen J (2018). Ephrin receptor A2 is a functional entry receptor for Epstein-Barr virus. Nat. Microbiol.

[14] Zhang H (2018). Ephrin receptor A2 is an epithelial cell receptor for Epstein-Barr virus entry. Nat. Microbiol.

[15] Chakraborty S, Veettil MV, Bottero V, Chandran B (2012). Kaposi’s sarcoma-associated herpesvirus interacts with EphrinA2 receptor to amplify signaling essential for productive infection. Proc. Natl Acad. Sci. USA.

[16] Hahn AS (2012). The ephrin receptor tyrosine kinase A2 is a cellular receptor for Kaposi’s sarcoma-associated herpesvirus. Nat. Med..

[17] TerBush, A. A., Hafkamp, F., Lee, H. J. & Coscoy, L. A Kaposi’s sarcoma-associated herpesvirus infection mechanism is independent of integrins alpha3beta1, alphaVbeta3, and alphaVbeta5. *J. Virol*. **92**, 10.1128/jvi.00803-18 (2018). 10.1128/JVI.00803-18 PMC6096800 29899108

[18] Chen, J., Zhang, X., Schaller, S., Jardetzky, T. S. & Longnecker, R. Ephrin receptor A4 is a new Kaposi’s sarcoma-associated herpesvirus virus entry receptor. *mBio* **10**, 10.1128/mBio.02892-18 (2019). 10.1128/mBio.02892-18 PMC6381284 30782663

[19] Grosskopf, A. K. et al. EphA7 functions as receptor on BJAB cells for cell-to-cell transmission of the Kaposi’s sarcoma-associated herpesvirus and for cell-free infection by the related rhesus monkey rhadinovirus. *J. Virol*. **93**, 10.1128/jvi.00064-19 (2019). 10.1128/JVI.00064-19 PMC6639272 31118261

[20] Wang J, Zheng X, Peng Q, Zhang X, Qin Z (2020). Eph receptors: the bridge linking host and virus. Cell Mol. Life Sci..

[21] Himanen JP (2009). Ligand recognition by A-class Eph receptors: crystal structures of the EphA2 ligand-binding domain and the EphA2/ephrin-A1 complex. EMBO Rep..

[22] Irie N (2009). Bidirectional signaling through ephrinA2-EphA2 enhances osteoclastogenesis and suppresses osteoblastogenesis. J. Biol. Chem..

[23] Himanen JP (2010). Architecture of Eph receptor clusters. Proc. Natl Acad. Sci. USA.

[24] Pasquale EB (2005). Eph receptor signalling casts a wide net on cell behaviour. Nat. Rev. Mol. Cell Biol..

[25] Pasquale EB (2008). Eph-ephrin bidirectional signaling in physiology and disease. Cell.

[26] Hahn AS, Desrosiers RC (2014). Binding of the Kaposi’s sarcoma-associated herpesvirus to the ephrin binding surface of the EphA2 receptor and its inhibition by a small molecule. J. Virol..

[27] Chowdary TK (2010). Crystal structure of the conserved herpesvirus fusion regulator complex gH-gL. Nat. Struct. Mol. Biol..

[28] Xing Y (2015). A site of varicella-zoster virus vulnerability identified by structural studies of neutralizing antibodies bound to the glycoprotein complex gHgL. Proc. Natl Acad. Sci. USA.

[29] Chandramouli, S. et al. Structural basis for potent antibody-mediated neutralization of human cytomegalovirus. *Sci. Immunol*. **2**, 10.1126/sciimmunol.aan1457 (2017). 10.1126/sciimmunol.aan1457 28783665

[30] Hahn AS, Desrosiers RC (2013). Rhesus monkey rhadinovirus uses eph family receptors for entry into B cells and endothelial cells but not fibroblasts. PLoS Pathog..

[31] Himanen JP (2004). Repelling class discrimination: ephrin-A5 binds to and activates EphB2 receptor signaling. Nat. Neurosci..

[32] Chrencik JE (2006). Structural and biophysical characterization of the EphB4*ephrinB2 protein-protein interaction and receptor specificity. J. Biol. Chem..

[33] Bowden TA (2009). Structural plasticity of eph receptor A4 facilitates cross-class ephrin signaling. Structure.

[34] Borza CM, Hutt-Fletcher LM (2002). Alternate replication in B cells and epithelial cells switches tropism of Epstein-Barr virus. Nat. Med..

[35] Wang X, Kenyon WJ, Li Q, Mullberg J, Hutt-Fletcher LM (1998). Epstein-Barr virus uses different complexes of glycoproteins gH and gL to infect B lymphocytes and epithelial cells. J. Virol..

[36] Chen, J., Rowe, C. L., Jardetzky, T. S. & Longnecker, R. The KGD motif of Epstein-Barr virus gH/gL is bifunctional, orchestrating infection of B cells and epithelial cells. *mBio* **3**, 10.1128/mBio.00290-11 (2012). 10.1128/mBio.00290-11 PMC3251506 22215569

[37] Zhang N (2011). Binding of herpes simplex virus glycoprotein D to nectin-1 exploits host cell adhesion. Nat. Commun..

[38] Lu G (2014). Crystal structure of herpes simplex virus 2 gD bound to nectin-1 reveals a conserved mode of receptor recognition. J. Virol..

[39] Otwinowski Z, Minor W (1997). [20] Processing of X-ray diffraction data collected in oscillation mode. Methods Enzymol..

[40] Read RJ (2001). Pushing the boundaries of molecular replacement with maximum likelihood. Acta Crystallogr. D. Biol. Crystallogr.

[41] Emsley P, Cowtan K (2004). Coot: model-building tools for molecular graphics. Acta Crystallogr. D. Biol. Crystallogr.

[42] Adams PD (2010). PHENIX: a comprehensive Python-based system for macromolecular structure solution. Acta Crystallogr. D. Biol. Crystallogr.

[43] Chen VB (2010). MolProbity: all-atom structure validation for macromolecular crystallography. Acta Crystallogr. D. Biol. Crystallogr.

[44] Pettersen EF (2004). UCSF Chimera–a visualization system for exploratory research and analysis. J. Comput. Chem..

[45] Li A (2017). Structural basis of nectin-1 recognition by pseudorabies virus glycoprotein D. PLoS Pathog..

[46] Stecher G, Tamura K, Kumar S (2020). Molecular evolutionary genetics analysis (MEGA) for macOS. Mol. Biol. Evol..

[47] Ashkenazy H (2016). ConSurf 2016: an improved methodology to estimate and visualize evolutionary conservation in macromolecules. Nucleic Acids Res..

