# Supplementary material for: Molecular basis of EphA2 recognition by gHgL from gammaherpesviruses
Source: Nat Commun. 2020 Nov 24;11:5964. doi: 10.1038/s41467-020-19617-9 (PMC7687889; doi:10.1038/s41467-020-19617-9)
Supplement: Supplementary file 1 — Supplementary Information [file 41467_2020_19617_MOESM1_ESM.pdf]

Supplementary Information

Molecular basis of EphA2 recognition by gHgL from gammaherpesviruses

Chao Su, et al

Chao Su, Lili Wu, Yan Chai, Jianxun Qi, Shuguang Tan, George F. Gao, Hao Song, Jinghua Yan

Supplementary Table 1. Data collection and refinement statistics

	KSHV gHgL-LBD	EBV gHgL-LBD
**Data collection**		
Space group	*P212121*	*P1*
Cell dimensions		
a, b, c (Å)	80.80, 153.20, 275.23	94.46, 113.81, 119.65
α , β, γ (°)	90.00, 90.00, 90.00	90.03, 90.17, 89.89
Resolution (Å)	50.00-3.20 (3.31-3.20)[a]	50.00-3.00 (3.11-3.00)[a]
Unique reflections	57447	98531
R_{merge}	0.134 (2.918)[a]	0.086 (1.102)[a]
R_{pim}	0.038 (0.813)[a]	0.054 (0.676)[a]
$I / \sigma I$	18.3 (0.989)[a]	12.950 (1.025)[a]
$CC_{1/2}$	0.997 (0.527)[a]	0.994 (0.757)[a]
Completeness (%)	99.6 (100)[a]	99.0 (98.6)[a]
Redundancy	12.9 (13.1)[a]	3.5 (3.6)[a]
**Refinement**		
Resolution (Å)	31.65-3.20	41.22-3.00
No. reflections	45163	98231
R_{work} / R_{free}	0.2393/0.2721	0.2587/0.2888
No. atoms		
Protein	14513	29096
Ligand/ion	388	224
Water	0	0
B-factors		
Protein	66.67	121.51
Ligand/ion	115.18	142.95
Water		
R.m.s. deviations		
Bond lengths (Å)	0.002	0.005
Bond angles (°)	0.477	1.08
Ramachandran plot		
Favored (%)	99.89	99.40
Allowed (%)	0.11	0.54
Outliers (%)	0.00	0.05

[a] Values in parentheses are for highest-resolution shell.

Supplementary Table 2. Interaction between LBD and EBV gHgL or KSHV gHgL

			Contacts[a]	LBD	Total contacts
EBV	gH	E30	1	P109	28
		G31	1, 1, 7	S107, F108, P109	
		H32	1, 10, 2, 5	V72, M73, F108, P109	
	gL	W24	2, 3, 3, 6, 1, 2	D53, M55, C70, R103 (1)[b], F108, C188	149
		A25	2, 4, 3, 1	S68, C70, C188, V189	
		Y26	2	N57	
		P27	8	R103	
		T32	1	I58	
		L34	3	I58	
		H38	3, 4	I58, P63	
		T55	4	R103	
		K68	2	D53 (1)	
		N72	1, 1, 2, 4	E40, L41, G42, L54	
		Q73	2,1	Y48, L54	
		L74	1, 17, 1, 1	E40, L54, M55, Y65	
		V75	8, 8, 3	L54 (1), M55, Q56 (1)	
		I76	6	Q56	
		S77	2, 10, 10	M55, Q56 (1), N57	
		R78	4, 4, 1	Q56 (1), I58, P63	
		R130	1	V161	
		A132	6, 1	M59, N60	
KSHV	gH	E52	7	R103	27
		F53	8, 3	R103, S107	
		N54	2	P109	
		FUC	7	P109	
	gL	Q20[c]	1, 13	C70, N71	290
		Y21	11, 1, 3, 3, 19, 2	C70, V72, M73, R103, F108, P109	
		V22	6, 6, 2	C70, R103 (1), C188	
		A23	2, 4, 3, 5, 2, 3	S68, C70, R103, C188, V189, A190	
		L24	1, 2, 3, 1	N57, M66, T101, A190	
		P25	9	R103	
		A28	1	M59	
		Q30	11, 7, 6, 11	I58, M59, N60 (1), D61 (1)	
		A31	7	D61	
		S32	4	D61 (1)	
		K61	2	N57	
		R63	5	M55	
		D68	1	L54	
		I69	5, 2, 3, 2	L54, M55, Q56, Y65	
		T70	6, 12, 5	L54 (1), M55, Q56 (1)	
		V71	12	Q56	
		E72	5, 6, 9, 1	M55, Q56 (1), N57 (1), I58	
		H127	3	M59	
		N128	14, 15	M59, N60(2)	
		V129	8, 4	M59, L163	
		N130	8, 11, 2	M59, N60, I64	

[a] Numbers represent the number of atom-to-atom contacts between the gHgL residues and LBD residues, which were analyzed by the contact program in CCP4 suite (the distance cutoff is 4.5 Å).
[b] Numbers in the parentheses represent the number of potential hydrogen bonds between the gHgL residues and LBD residues.
[c] protein-expression vector-introduced extra residue.

Supplementary Table 3. The accession numbers for gH and gL proteins used for alignment

Organism	Abbreviation	Accession No. (gH)	Accession No. (gL)	Accession No. (gB)
**Kaposi's sarcoma-associated herpesvirus**	KSHV	YP_001129375.1	YP_001129399.1	YP_001129354.1
**Epstein-Barr virus**	EBV	YP_401700.1	YP_401678.1	YP_401713.1
**Rhesus monkey rhadinovirus**	RRV	AAF60000.1	AAF60026.1	
**Alcelaphine gammaherpesvirus 1**	AlHV-1	NP_065521.1	NP_065545.1	NP_065511.1
**Alcelaphine gammaherpesvirus 2**	AlHV-2	YP_009044405.1	YP_009044429.1	
**Bovine gammaherpesvirus 6**	BoHV-6	YP_009042001.1	YP_009042024.1	
**Ovine gammaherpesvirus 2**	OvHV-2	YP_438146.1	YP_438169.1	
**Porcine lymphotropic herpesvirus 1**	PLHV-1	YP_009505348.1	YP_009505372.1	
**Ateline gammaherpesvirus 3**	AtHV-3	NP_047993.1	NP_048020.1	
**Bovine gammaherpesvirus 4**	BoHV-4	NP_076514.1	NP_076539.1	
**Cricetid gammaherpesvirus 2**	CrGHV-2	YP_004207858	YP_004207883.1	
**Retroperitoneal fibromatosis-associated herpesvirus**	RFHVMm	AGY30705.1	AGY30728.1	
**Macaca fuscata rhadinovirus**	MFRV	AAT00015.1	AAT00055.1	
**Macaca nemestrina rhadinovirus 2**	MNRV-2	AJE29663.1	AJE29691.1	
**Murid gammaherpesvirus 4**	MuHV-4	NP_044860.1	AAF19311.1	NP_044848.3
**Wood mouse herpesvirus**	WMHV	ACY41096.1	ACY41118.1	
**Saimiriine gammaherpesvirus 2**	SaHV-2	NP_040224.1	NP_040249.1	
**Macacine gammaherpesvirus 5**	MaHV-5	NP_570762.1	NP_570788.1	
**Common bottlenose dolphin gammaherpesvirus 1 strain Sarasota**	CBDHV-1	YP_009388523.1	YP_009388547.1	
**Callitrichine gammaherpesvirus 3**	CaHV-3	NP_733867.1	NP_733891.1	
**Rhesus lymphocryptovirus**	rhLCV	YP_067997.1	YP_067974.1	
**Lymphocryptovirus Macaca/pfe-lcl-E3**	LCVMa	ALF03266.1	ALF03246.1	
**Equid gammaherpesvirus 2**	EHV-2	NP_042618.1	NP_042644.1	NP_042604.1
**Equid gammaherpesvirus 5**	EHV-5	YP_009118412.1	YP_009118436.1	
**Felis catus gammaherpesvirus 1**	FcaGHV1	YP_009173898.1	YP_009173923.1	
**Harp seal herpesvirus**	HSHV	AJG42948.1	AJG42971.1	
**Eptesicus fuscus gammaherpesvirus**	EfHV	YP_009552485.1	YP_009552510.1	
**Rhinolophus gammaherpesvirus 1**	RGHV-1	YP_009551832.1	YP_009551856.1	
**Myotis gammaherpesvirus 8**	MyHV-8	YP_009229856.1	YP_009229879.1	
**Human herpesvirus 1**	HSV-1	YP_009137096.1	YP_009137075.1	

**Human herpesvirus 2**	HSV-2	YP_009137173.1	YP_009137152.1	YP_009137179.1
**Varicella-zoster virus**	VZV	NP_040160.1	NP_040182.1	
**Human cytomegalovirus**	HCMV	YP_081523.1	YP_081555.1	
**Human herpesvirus 6a**	HHV-6A	NP_042941.1	NP_042975.1	
**Human herpesvirus 6b**	HHV-6B	NP_050229.1	NP_050261.1	
**Human herpesvirus 7**	HHV-7	YP_073788.1	YP_073820.1	

Supplementary Figure

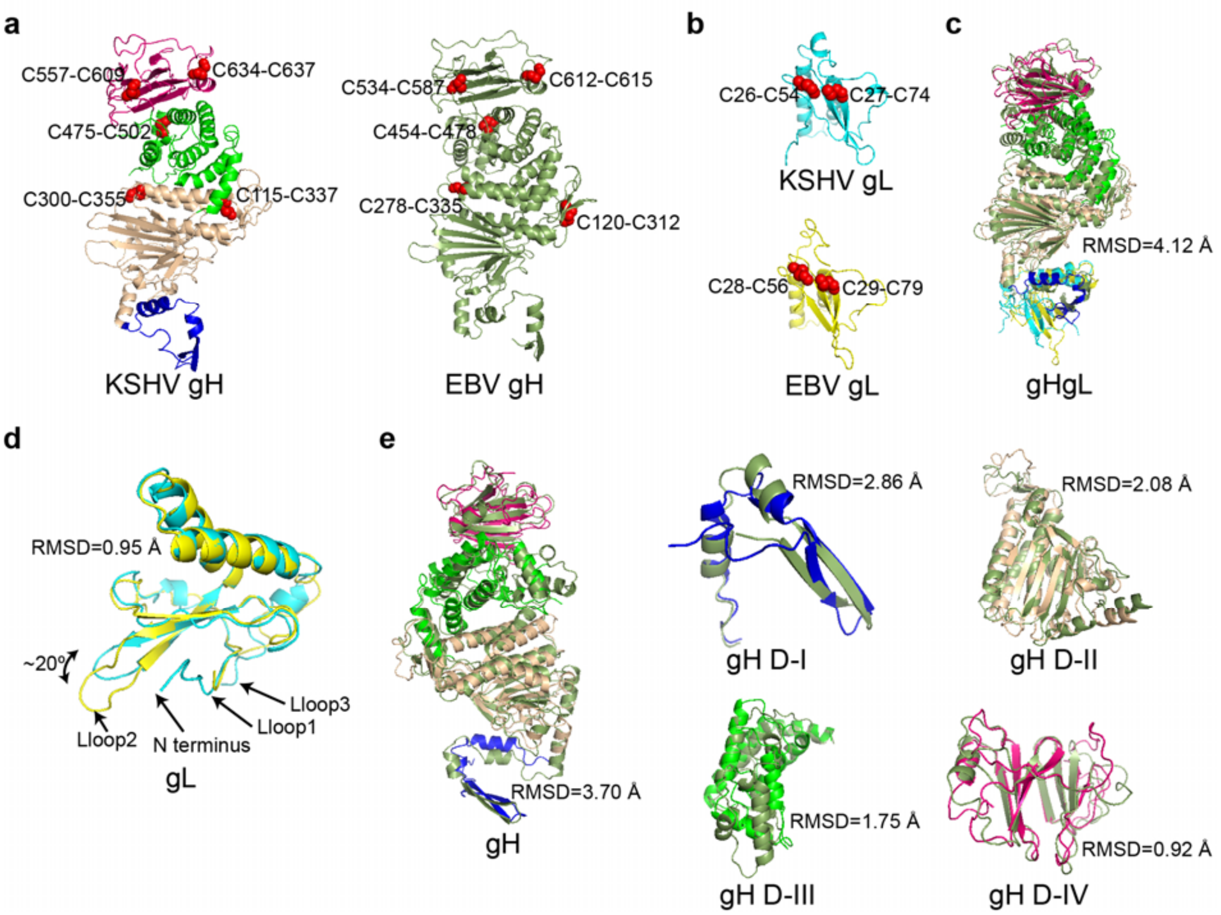

Supplementary Figure 1. Structural comparison between KSHV gHgL and EBV gHgL. a,**b**, Cartoon diagram of gH (**a**) or gL (**b**) of KSHV and EBV. The disulfide bonds are shown. **c**-**e**, Superimposition of gHgL (**c**), gL (**d**), gH and its domains (**e**) of KSHV with that of EBV. KSHV gHgL and EBV gHgL are colored as Fig. 2**a**. RMSD values are labeled.

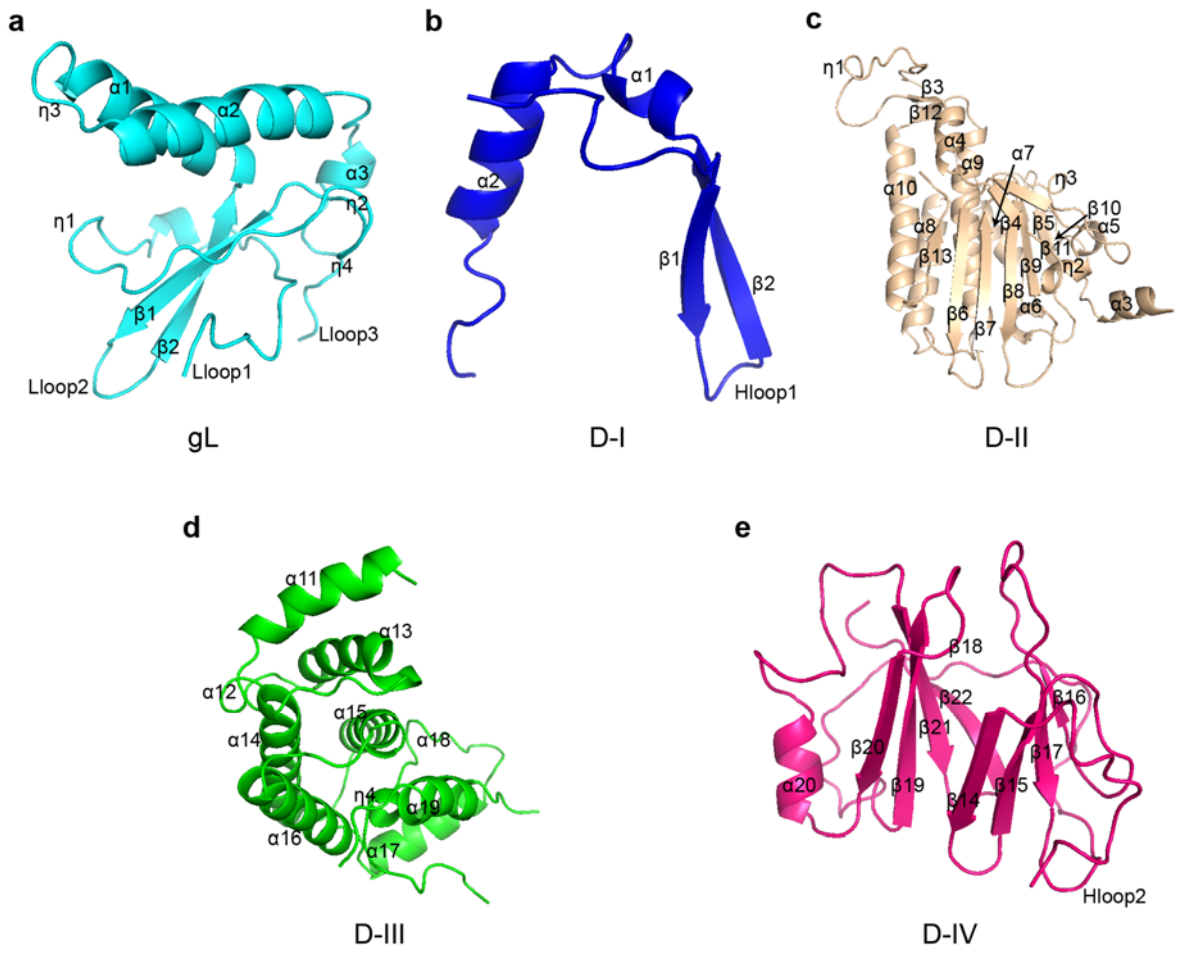

Supplementary Figure 2. Cartoon diagram of domains of KSHV gH and gL structure. a-e, The secondary structure elements are labeled. gH and gL loop shown are indicated with a preceding H or L, respectively. Ribbon diagram of gL (**a**), gH domain I (D-I) (**b**), gH domain II (D-II) (**c**), gH domain III (D-III) (**d**) and gH domain IV (D-IV) (**e**) are shown as in Fig. 2**a**.

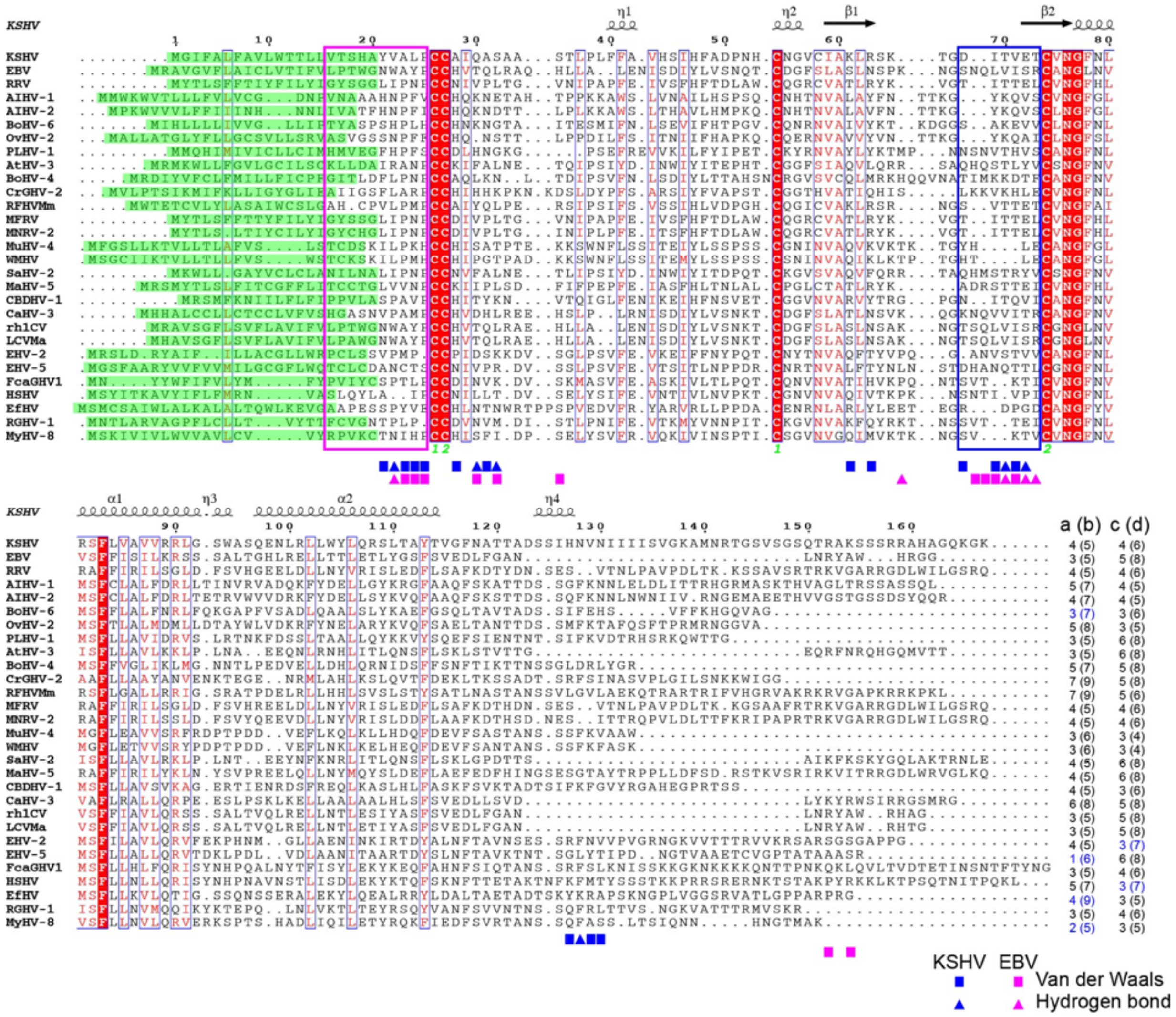

Supplementary Figure 3. Sequence alignment of gL proteins from 29 representative γ-herpesviruses. The blue or magenta triangles indicate LBD-binding amino acids via hydrogen bonds in KSHV gL or EBV gL, respectively. The blue or magenta squares indicate amino acids that contribute to binding to LBD via Van der Waals in KSHV gL or EBV gL, respectively. The predicted signal peptide produced by SignalP-5.0 Server[1] is shown by green rectangles. Magenta and blue rectangle represent two regions that bind to LBD, respectively. In the first region, b and a are represented the number of all residues and hydrophobic residues from the N-terminus to the first conserved cysteine residue, respectively. In the second region, d and c are represented the number of all residues and hydrophilic residues before the fourth conserved cysteine residue, respectively. The ratio of a to b or c to d less than 0.5 are highlighted in blue. Sequence alignment

was produced by ESPript[2]. The GenBank accession codes are shown in the Supplementary Table 3.

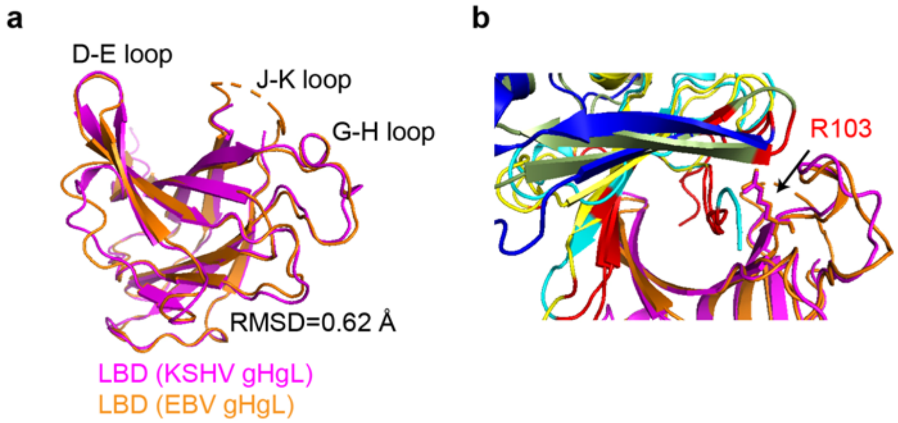

Supplementary Figure 4. Structural comparison between KSHV gHgL-LBD and EBV gHgL-LBD. **a**, Superimposition of LBDs in KSHV gHgL-LBD and EBV gHgL-LBD. LBDs in EBV gHgL-LBD and KSHV gHgL-LBD are colored orange and magenta, respectively. D-E, G-H and J-K loop, and RMSD are shown. **b**, R103 in the channel of LBD is highlighted in stick.

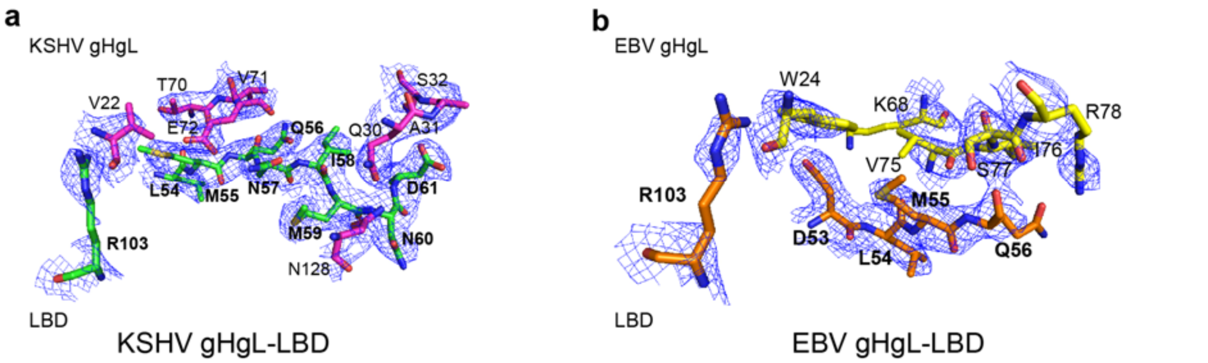

Supplementary Figure 5. Representative electron density maps for the two complex structures. a,**b**, The final 2Fo-Fc density maps of the complex structure of LBD bound to KSHV gHgL (**a**) or EBV gHgL (**b**) are drawn in blue mesh contoured at 1 sigma, respectively.

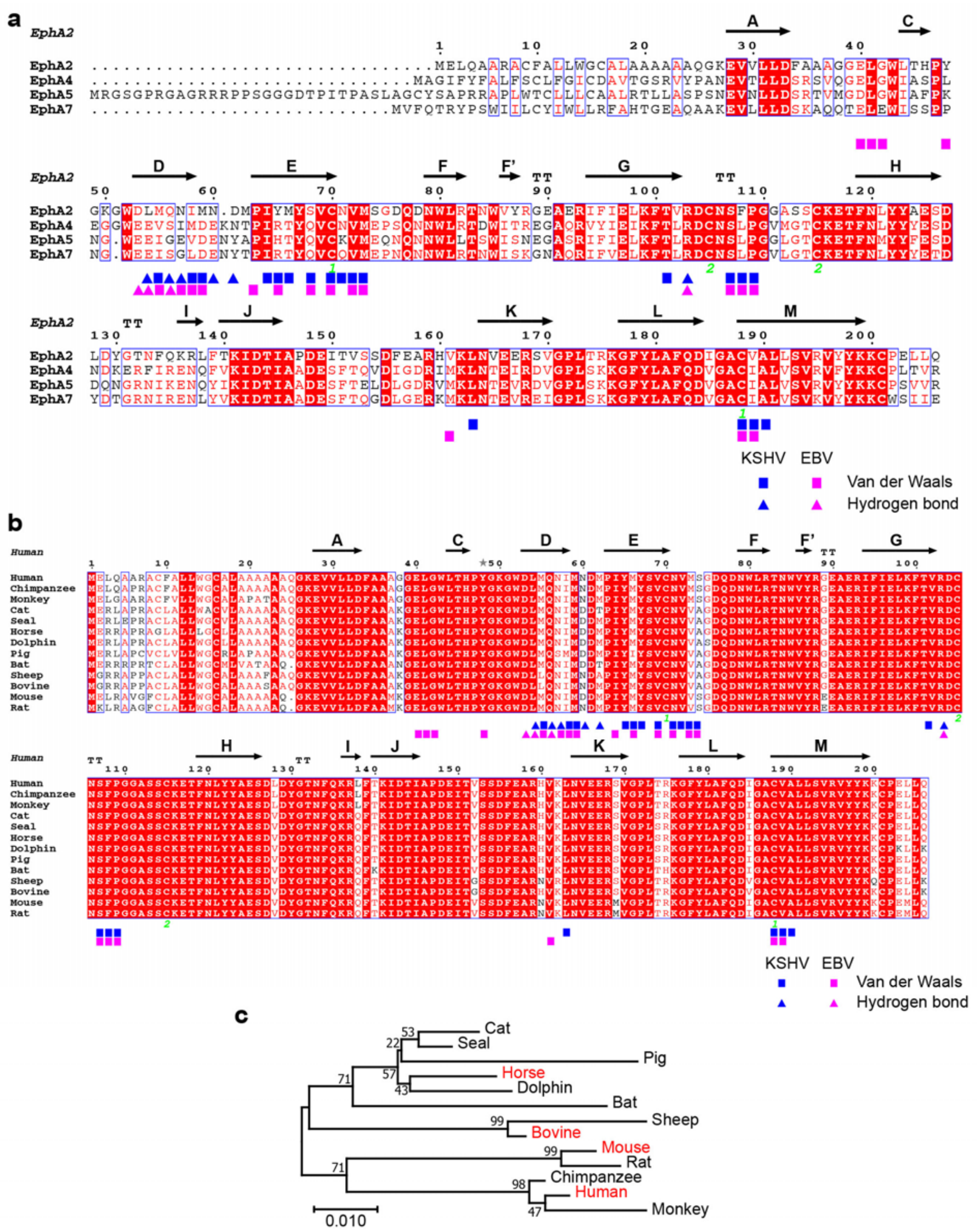

Supplementary Figure 6. Sequence alignment of EphAs. a, Sequence alignment of LBDs from human EphA2, EphA4, EphA5 and EphA7 proteins. The blue or magenta triangles indicate amino

acids in LBD that bind to KSHV gHgL or EBV gHgL via hydrogen bonds. The blue or magenta squares indicate amino acids in LBD that contribute to bind to KSHV gHgL or EBV gHgL via Van der Waals. Sequence alignment was produced by ESPript[2]. The GenBank accession codes: EphA4, AAH26327.1; EphA5, AAI43428.1; EphA7, NP_001363394.1. **b**, Sequence alignment of EphA2 LBD from different mammals. The GenBank accession codes: Chimpanzee, XP_016810290.1; Monkey, NP_001035768.1; Cat, XP_023113942.1; Seal, XP_021539729.1; Horse, XP_001488789.1; Dolphin, XP_026965844.1; Pig, XP_005665094.1; Bat, XP_006105814.2; Sheep, XP_027821486.1; Bovine, NP_001192660.1; Mouse, NP_034269.2; Rat, NP_001102447.1. **c**, Phylogenetic tree analysis of EphA2 LBD from different mammals. The host species infected by the selected viruses including AIHV-1, EHV-2 and MuHV-4 for experiment are highlighted.

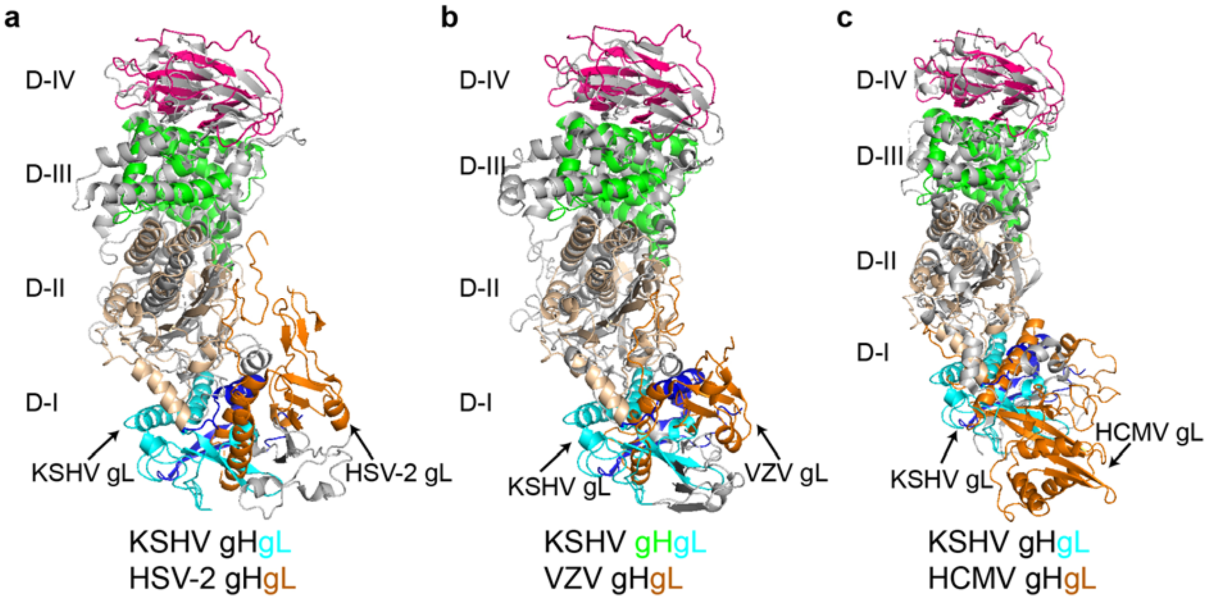

Supplementary Figure 7. Structural comparison between KSHV gHgL and gHgL from α- and β-herpesvirus. a-**c**, Superimposition of KSHV gHgL and HSV-2 gHgL (PDB: 3M1C) (**a**) or VZV gHgL (PDB: 4XHJ) (**b**) or HCMV gHgL (PDB: 5VOB) (**c**). KSHV gH and gL are colored as in Fig. 2**a**. gH and gL of HSV-2, VZV and HCMV are colored gray and orange, respectively.

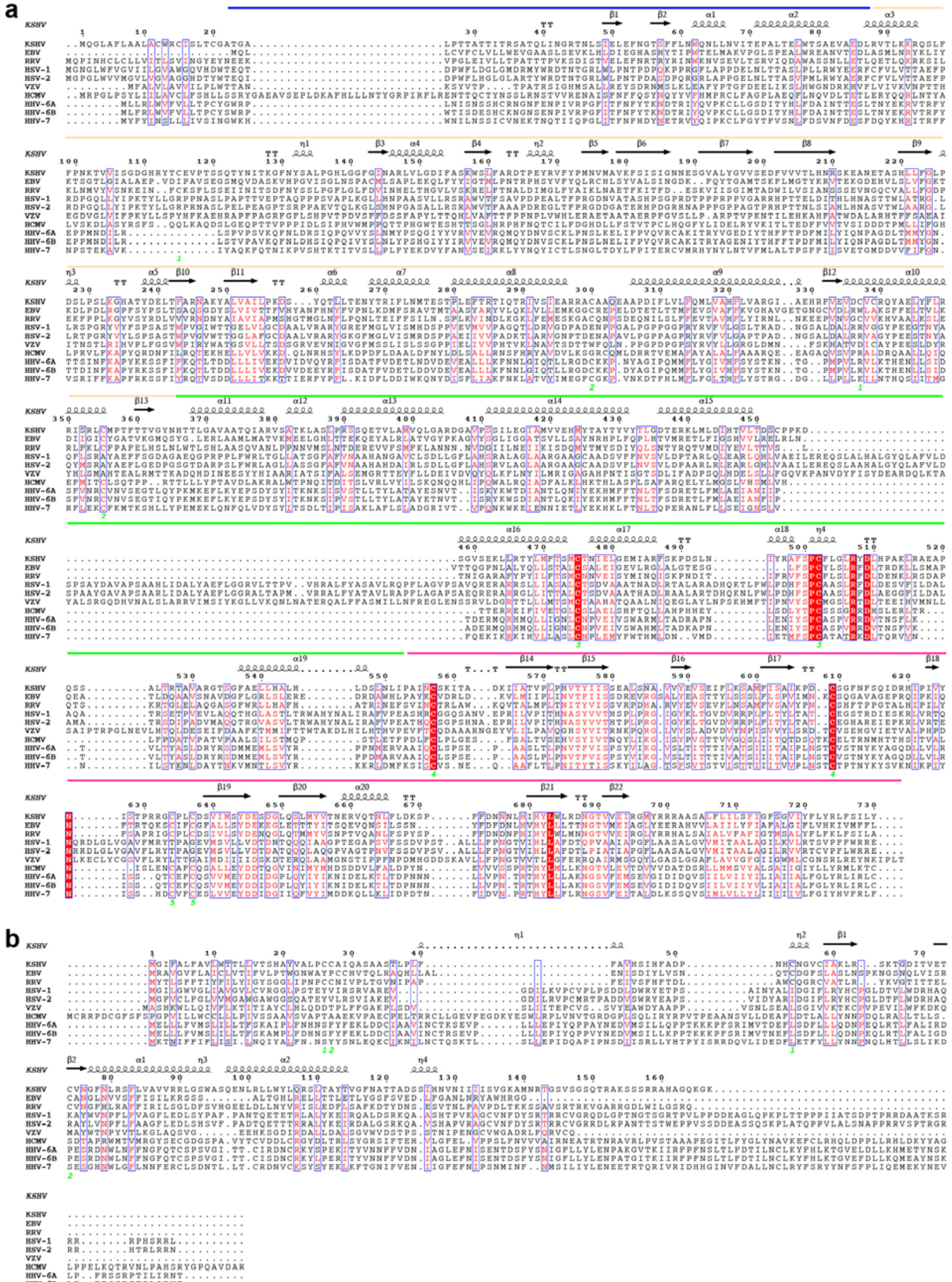
a
b
KSHV
EBV
RRV
HSV-1
HSV-2
VZV
HCMV
HHV-6A
HHV-6B
HHV-7

Supplementary Figure 8. Sequence alignment of gHgL proteins from 10 representative herpesviruses. **a**,**b**, gH (**a**) and gL (**b**) proteins from representative α-, β- and γ-herpesvirus are included for alignment. Different colored lines are corresponding to the different domains of gH. Sequence alignment was produced by ESPript[2]. The GenBank accession codes are shown in the Supplementary Table 3.

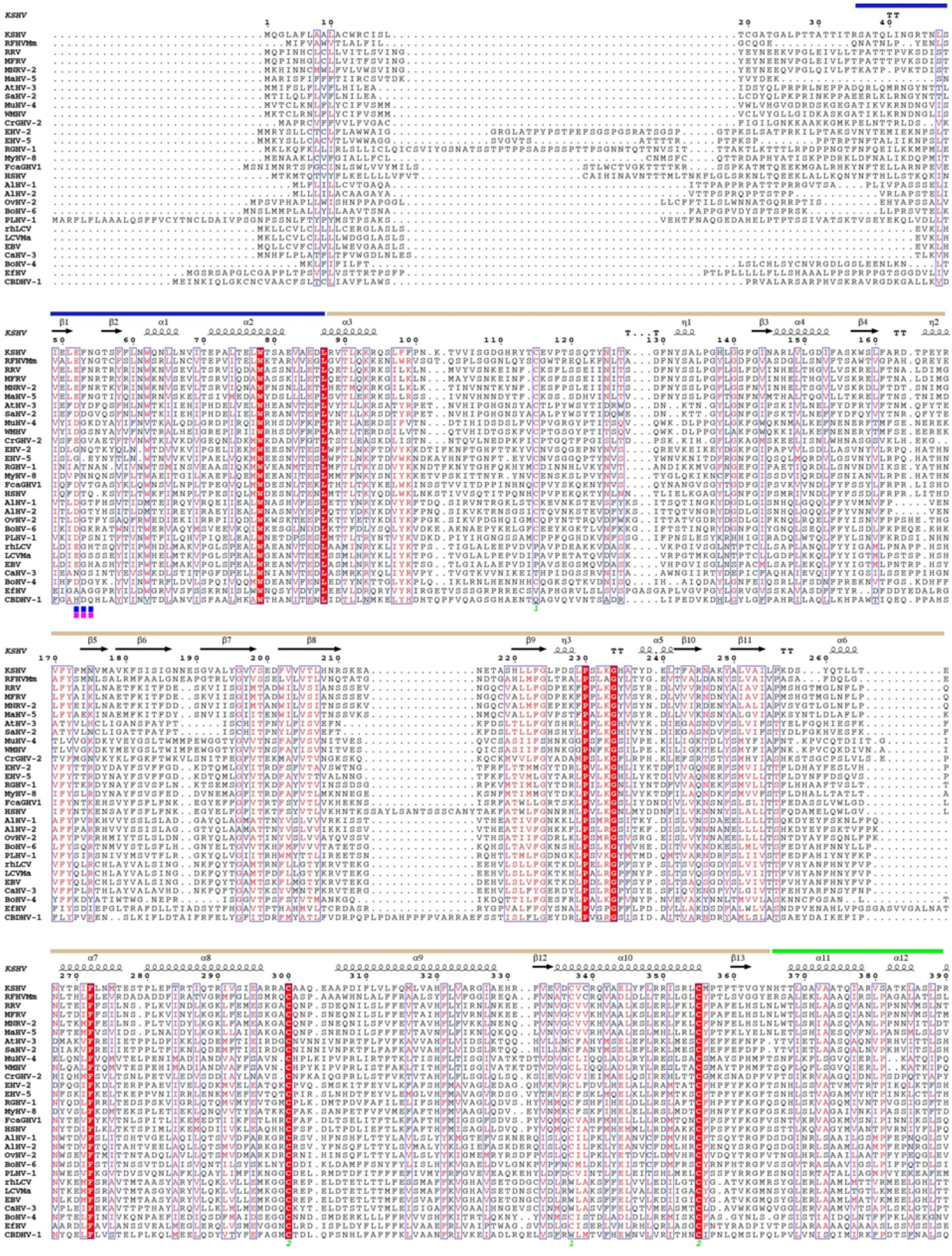
KSHV
RFHVMm
RRV
MFRV
MNRV-2
MaHV-5
AtHV-3
SaHV-2
MuHV-4
WMHV
CrGHV-2
EHV-2
EHV-5
RGHV-1
MyHV-8
FcaGHV1
HSHV
AlHV-1
AlHV-2
OvHV-2
BoHV-6
PLHV-1
rhLCV
LCVMa
EBV
CaHV-3
BoHV-4
EfHV
CBDHV-1

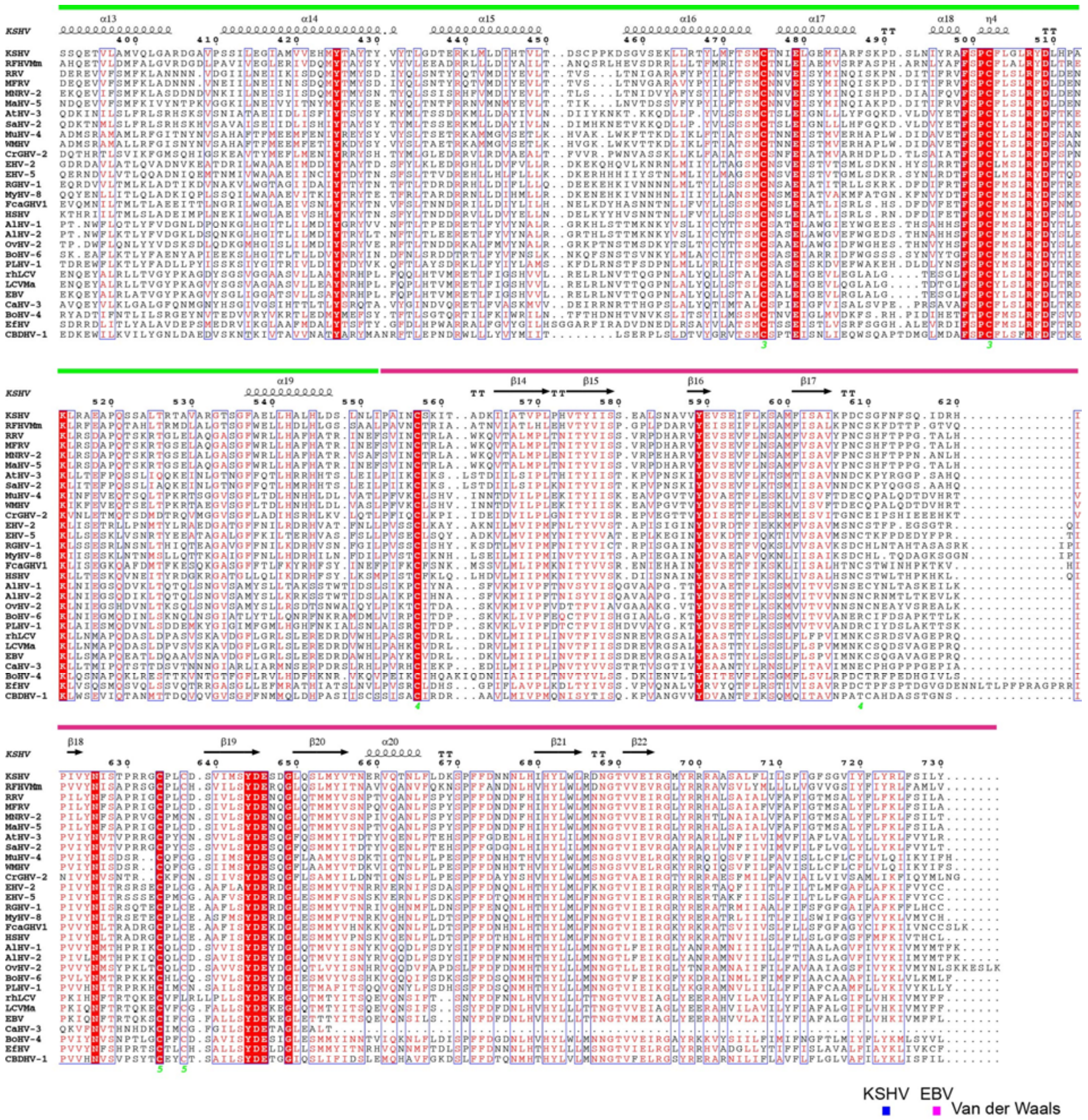

Supplementary Figure 9. Sequence alignment of gH proteins from 29 representative γ-herpesviruses. The blue or magenta squares indicate the amino acids contributing to LBD interaction via Van der Waals from KSHV or EBV, respectively. Different colored lines are corresponding to the different domains of gH. Sequence alignment was produced by ESPript[2]. The GenBank accession codes are shown in the Supplementary Table 3.

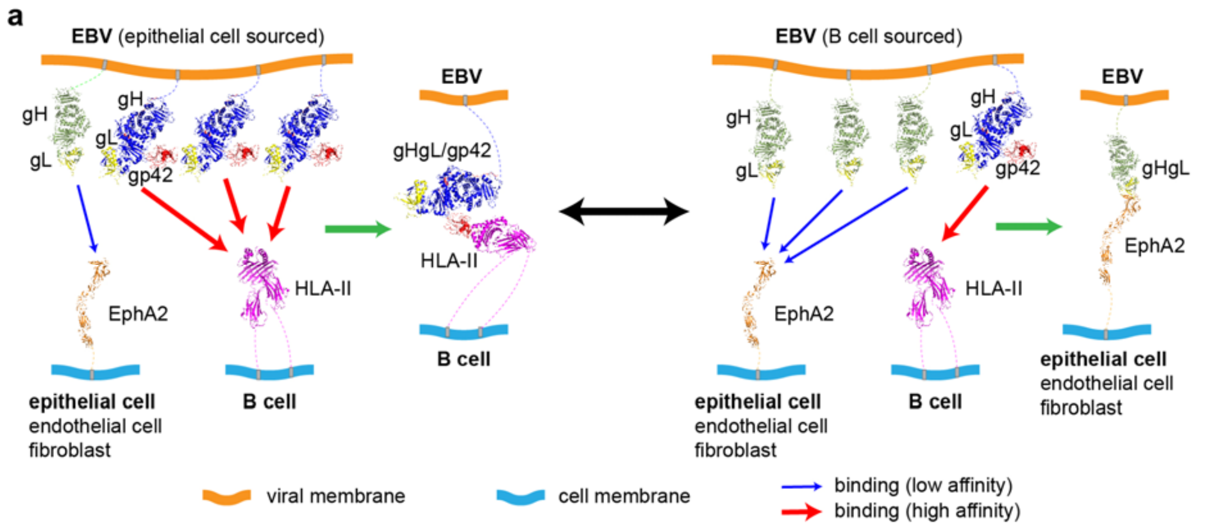

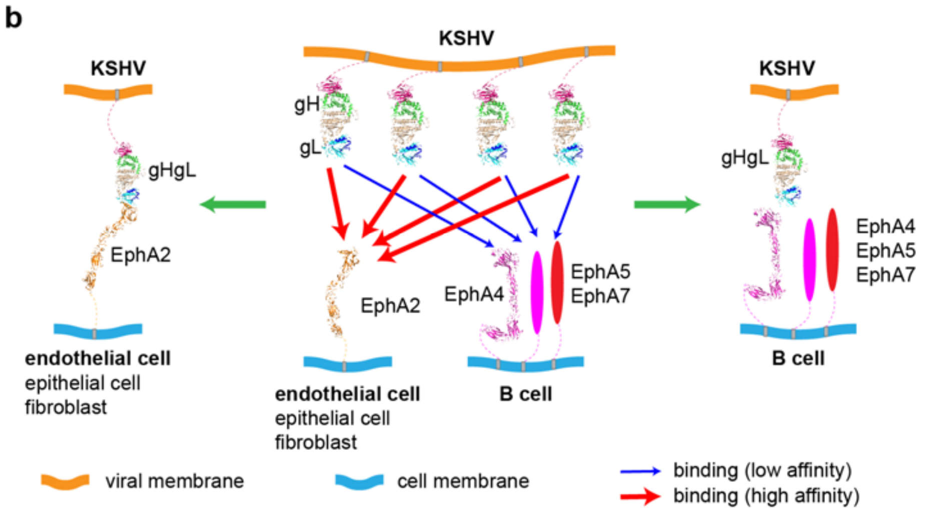

Supplementary Figure 10. Modes of EBV and KSHV binding to high- and low-affinity receptors for entry. a, Epithelial cell-derived EBV virions contain more gHgL-gp42 complex than gHgL complex. Although gHgL complex can bind to low-affinity receptor EphA2 in the epithelial cell, the higher binding of gHgL-gp42 to high-affinity receptor HLA-II in the B cell make virions better infect B cells. Virions originating in B cells contain more gHgL complex due to the degradation of gp42 in the cells. Enough gHgL proteins binding to low-affinity receptor EphA2 enable virions to infect epithelial cells effectively. Therefore, EBV uses this strategy to alter and switch the tropism of virus[3]. The structural views of HLA-II (PDB: 1KG0), EBV gHgL-gp42 (PDB: 5T1D), EphA2 (PDB: 2X10) and the complexes of EBV gHgL-gp42-HLA-II and EBV gHgL-EphA2 were generated using PyMOL software. **b**, KSHV virions bind to the high-

affinity receptor EphA2 for entry into endothelial cells. In addition, KSHV could bind to the low-affinity receptors in B cell, such as EphA4, EphA5 and EphA7. Multiple types of low-affinity receptors in B cell make sure the infection efficiency of KSHV. The structural views of EphA2 (PDB: 2X10), EphA4 (PDB: 4M4P) and the complexes of KSHV gHgL-EphA2 were generated using PyMOL software.

Supplementary Reference

1 Almagro Armenteros, J. J. *et al.* SignalP 5.0 improves signal peptide predictions using deep neural networks. *Nat Biotechnol* **37**, 420-423, doi:10.1038/s41587-019-0036-z (2019).

2 Robert, X. & Gouet, P. Deciphering key features in protein structures with the new ENDscript server. *Nucleic Acids Res* **42**, W320-324, doi:10.1093/nar/gku316 (2014).

3 Borza, C. M. & Hutt-Fletcher, L. M. Alternate replication in B cells and epithelial cells switches tropism of Epstein-Barr virus. *Nature medicine* **8**, 594-599, doi:10.1038/nm0602-594 (2002).
