# Supplementary material for: Molecular basis of EphA2 recognition by gHgL from gammaherpesviruses
Source: Nat Commun. 2020 Nov 24;11:5964. doi: 10.1038/s41467-020-19617-9 (PMC7687889; doi:10.1038/s41467-020-19617-9)
Supplement: Supplementary file 3 — Reporting Summary [file 41467_2020_19617_MOESM3_ESM.pdf]

nature research

Corresponding author(s): George F. Gao

Last updated by author(s): Oct 16, 2020

Reporting Summary

Nature Research wishes to improve the reproducibility of the work that we publish. This form provides structure for consistency and transparency in reporting. For further information on Nature Research policies, see our Editorial Policies and the Editorial Policy Checklist.

Statistics

For all statistical analyses, confirm that the following items are present in the figure legend, table legend, main text, or Methods section.

n/a	Confirmed	
☐	☒	The exact sample size (n) for each experimental group/condition, given as a discrete number and unit of measurement
☐	☒	A statement on whether measurements were taken from distinct samples or whether the same sample was measured repeatedly
☐	☒	The statistical test(s) used AND whether they are one- or two-sided<br>*Only common tests should be described solely by name; describe more complex techniques in the Methods section.*
☒	☐	A description of all covariates tested
☒	☐	A description of any assumptions or corrections, such as tests of normality and adjustment for multiple comparisons
☐	☒	A full description of the statistical parameters including central tendency (e.g. means) or other basic estimates (e.g. regression coefficient) AND variation (e.g. standard deviation) or associated estimates of uncertainty (e.g. confidence intervals)
☐	☒	For null hypothesis testing, the test statistic (e.g. F, t, r) with confidence intervals, effect sizes, degrees of freedom and P value noted<br>*Give P values as exact values whenever suitable.*
☒	☐	For Bayesian analysis, information on the choice of priors and Markov chain Monte Carlo settings
☒	☐	For hierarchical and complex designs, identification of the appropriate level for tests and full reporting of outcomes
☒	☐	Estimates of effect sizes (e.g. Cohen's d, Pearson's r), indicating how they were calculated

Our web collection on statistics for biologists contains articles on many of the points above.

Software and code

Policy information about availability of computer code

Data collection	X-ray diffraction data of KSHV gHgL-LBD complex were collected at Shanghai Synchrotron Radiation Facility (SSRF) BL17U, while the data of EBV gHgL-LBD complex were collected at BL19U. The Data were collected and processed by HKL2000. The firefly luciferase and Renilla luciferase activities were detected using a GloMax-96 Microplate Luminometer, and collected by Microsoft Excel 2016.
Data analysis	Data analysis in structural study: Phenix 1.16, Phaser, CCP4 7.1, HKL2000, Coot 0.8.9, MolProbity, UCSF Chimera 1.14, PyMOL 2.3.2. Data analysis in functional assays: Biacore Insight Evaluation 1.0.5.11069, GraphPad Prism 8, OriginPro 8.5. Phylogenetic tree analysis: MEGA 10.1.

For manuscripts utilizing custom algorithms or software that are central to the research but not yet described in published literature, software must be made available to editors and reviewers. We strongly encourage code deposition in a community repository (e.g. GitHub). See the Nature Research guidelines for submitting code & software for further information.

Data

Policy information about availability of data

All manuscripts must include a data availability statement. This statement should provide the following information, where applicable:

- Accession codes, unique identifiers, or web links for publicly available datasets
- A list of figures that have associated raw data
- A description of any restrictions on data availability

The structural data that support the findings of this study have been deposited in the Protein Data Bank. The accession numbers for the atomic coordinates and diffraction data reported in this paper are PDB: 7CZE (crystal structure of EphA2 LBD with EBV gHgL complex) and 7CZF (crystal structure of EphA2 LBD with KSHV gHgL complex). Figures 1a, 1b, 1c, 1d, 4b, 4c and 6d have associated raw data in this paper. The data that support the findings of this study are available from the corresponding author upon reasonable request.

April 2020

Field-specific reporting

Please select the one below that is the best fit for your research. If you are not sure, read the appropriate sections before making your selection.

- [x] Life sciences
- [ ] Behavioural & social sciences
- [ ] Ecological, evolutionary & environmental sciences

For a reference copy of the document with all sections, see nature.com/documents/nr-reporting-summary-flat.pdf

Life sciences study design

All studies must disclose on these points even when the disclosure is negative.

Sample size	Three independent biological replicates were used in this study because it is common in the biological experiments. No statistical methods were used to predetermine sample size. Sample sizes were chosen based on prior knowledge in the respective experiments and their intrinsic variability as performed in previous studies (Chen, J. et al. Nat Microbiol, 2018).
Data exclusions	No data were excluded from the analysis.
Replication	Experiments were repeated at least to n=3 to verify reproducibility. All attempts at replication were successful.
Randomization	For transfections, the WT and different mutants were allocated in different sequence of plates.
Blinding	For the cell fusion assay, when collecting the data, investigators were blinded to the group. Because samples were marked as numbers, only after checking the transfection sheet, the group allocation were known. For other experiments, the investigators were not blinded to group allocation during data collection or analysis. Because blinding has no effect on the experimental results.

Reporting for specific materials, systems and methods

We require information from authors about some types of materials, experimental systems and methods used in many studies. Here, indicate whether each material, system or method listed is relevant to your study. If you are not sure if a list item applies to your research, read the appropriate section before selecting a response.

Materials & experimental systems

n/a	Involved in the study
[x]	[ ] Antibodies
[ ]	[x] Eukaryotic cell lines
[x]	[ ] Palaeontology and archaeology
[x]	[ ] Animals and other organisms
[x]	[ ] Human research participants
[x]	[ ] Clinical data
[x]	[ ] Dual use research of concern

Methods

n/a	Involved in the study
[x]	[ ] ChIP-seq
[x]	[ ] Flow cytometry
[x]	[ ] MRI-based neuroimaging

Eukaryotic cell lines

Policy information about cell lines

Cell line source(s)	Sf9 and High Five insect cell line were from Invitrogen; HEK-293T cell (ATCC CRL-3216) were from ATCC.
Authentication	No cell authentication method was used.
Mycoplasma contamination	All cell lines tested negative for mycoplasma.
Commonly misidentified lines (See ICLAC register)	No misidentified lines were used.
