# Supplementary material for: Molecular basis of EphA2 recognition by gHgL from gammaherpesviruses
Source: Nat Commun. 2020 Nov 24;11:5964. doi: 10.1038/s41467-020-19617-9 (PMC7687889; doi:10.1038/s41467-020-19617-9)
Supplement: Supplementary file 2 — Peer Review [file 41467_2020_19617_MOESM2_ESM.pdf]

Reviewer #1 (Remarks to the Author):

The study deals with an interesting new field in the receptor research, the mechanism of host cell entry utilized by various viruses. The experiments in the manuscript are well executed and results properly discussed. Although Eph receptors and ephrins have been, by now, shown to be the entry receptors for several viruses (Henipa viruses, in particular), detailed studies of the entry mechanism are scarce. Thus, this study should be of interest for both Eph receptor investigators and 'functional virologists'.

The study reports crystal structures of Kaposi sarcoma-associated herpesvirus (KSHV) and Epstein-Barr virus attachment proteins in complex with the ligand-binding domain of EphA2. It shows, for the first time, how the viruses utilize a similar binding/recognizing mechanism as the biological EphA2 ligand, ephrin-A1. Interestingly, this mechanism is somewhat similar to the Henipa viruses that use ephrins as entry receptors. The study also demonstrates how the entry mechanism can be manipulated by structure-driven point mutations. Furthermore, the manuscript describes how a specific antibody can inhibit the entry of a virus to epithelial cells.

Thus, the manuscript warrants publication once certain changes have been implemented.

The main issue is language: there are far too many cases to list here but, as an example, a sentence like "Both total of 25 amino acids in LBD were observed…" has to be seriously rewritten and, overall, the whole manuscript has to go through a serious editing by an independent language reviewer

Other points:
1. Since the Kd of EphA2/LBD binding to EBV/gHgL is 26.3 μM, the authors must emphasize in the methods that the concentration of EphA2/EBV (10 mg/ml) was clearly above this value.
2. Does the difference in the number of glycosylation sites (page 5) in the two gHgL proteins have any biological relevance? If not, omit the sentence on page 5.
3. "…gL interacts with the 'peripheral region' of LBD…" (page 6) – explain peripheral region in more detail.
4. A surface area of 1,180 Å2 is barely 'larger' than 994 Å2 (page 7) – don't use the word 'larger'.
5. "…the hydrogen bond interaction formed by R103 is scrucial…" (page 9) – name the binding partners.
6. "…residues in the core secondary structure of KSHV and EBV gL are more conserved…" – be precise, more than what?
7. The last paragraph of the results (page 11) – the authors are running through this paragraph; please explain the experiments and the results more elaborately.
8. Figure 1c – the figure doesn't show a shift in the elution volume between EBV-gHgL and EBV-gHgL/LBD

Reviewer #2 (Remarks to the Author):

Su et al. present crystal structures of the envelope glycoprotein complexes gHgL from two oncogenic human herpesviruses, Epstein–Barr virus (EBV) and Kaposi's sarcoma-associated herpesvirus (KSHV), in complex with the ligand-binding domain (LBD) of the ephrin receptor tyrosine kinase A2 (EphA2). Both viruses have a tropism, namely, which cell types that they can infect, that is not fully understood. EBV infects human B cells and epithelial cells. The B cell infection pathway is better characterized and a crystal structure of a gHgL/gp42 complex has been reported (see reference 18). Recently, two independent groups identified EphA2 as a possible receptor for EBV during infection of epithelial cells (see references 7 and 8). Su et al. now define the molecular basis for this interaction. They visualize how gHgL of both viruses binds the LBD of EphA2 in a conserved manner. The authors' careful comparison of the binding interfaces provides plausible explanations for their measured

differential affinities of KSHV or EBV gHgL to the host cell receptor domain. As one would predict from their structures, mutations of critical binding interface residues to alanine negatively affects fusion efficiency in a cell-based assay. From the structural interpretation, it is evident that engaging EphA2 by gHgL, a previously reported neutralizing antibody E1D1, or its native ligand is mutually exclusive. The authors conclude their study by showing that envelope glycoproteins from other herpesvirus genera, after comparing their structures to KSHV, are able to promote cell fusion as well when partnered with EphA2.

The manuscript is well presented with well-designed figures that depict the structural results, and an appropriate interpretation of the results. Sufficient details of the methodology are explained. This study is a significant step forward in our advancement of understanding the pathology of herpesviruses. I do have a few comments.

Major comments:

The English contains many errors and needs editing. Please get help from an editor or native English speaker before resubmitting.

Previously determined crystal structures of gHgL should be referenced in the introduction.

It is true that on and off rate constants are in principle measured in the surface plasmon resonance (SRP) assays, but the authors only present calculated affinity constants. Instead of writing “quantify the kinetics” (line 76) it would be better to write “measure affinity constants”.

The reported unit cell angles for the EBV gHgL-LBD crystal structure are 90.03, 90.167, 89.888 degrees, which is very close to 90 degrees. Can the authors comment on why they assigned space group P1. Does the data not scale in a higher symmetry spacegroup, e.g. it appears that the data can the indexed on a primitive monoclinic or orthorhombic Bravais lattice? How many molecules are in the asymmetric units of the two crystals? Have the authors analyzed the datasets for possible crystal twinning?

How well are the N-linked glycans resolved? Did the authors observe electron density for at least the first NAG at each of the positions that is labeled red in Fig. 2b?

In general the figures are excellent and allowed me to understand the science presented very well on their own, something I really appreciate. Ideally, the authors would choose the same color scheme -- at least for the EBV structure -- which was already used for coloring the domains D-I, D-II, D-III and D-IV in the previous paper published in this journal (reference 18). The coloring of the domains in Fig. 2b definitely needs to be changed to make it consistent with the colors used in the other figure panels. For instance, in Fig. 2b domain D-III is yellow, but in Fig. 2a an LBD is yellow, which is confusing. After looking at Fig. S1, I’m not convinced that it is a good idea to have 2 different coloring schemes, one for Fig. 2a, c-f, and a different one for Fig. 2b and S1. I’m also not sure whether it is necessary to have different colors for the KSHV and EBV structures. The way the authors did it in Fig. S1 (color one in gray if superposed) works very well.

Fig. 2c,f, please define which residues were used for calculating the superposition transformation matrix. Only residues of LBD?

I would replace the right panel of Fig. 2c with Fig. S5b.

Simulated annealing omit maps covering the regions shown in Fig. 3e and f should be provided as a supplementary figure. This would allow one to judge how accurate the models might be defined at the binding interface.

In Fig. 3, if I understand correctly, the definition of overlapping residues is not same in Fig. 3c-d (LBD residues that bind gH and gL) and in Fig. 3g (LBD residues that bind KSHV and EBV). This is confusing.

Minor comments:

Line 18, please define gHgL in the abstract, e.g. "envelope glycoproteins".

Line 22, line 64, line 237, the term "network" should not be used here. A network is a system where information is transferred between nodes and therefore an inappropriate term to describe molecular contacts of a protein-protein binding interface.

Line 25, "proved" is too strong here. "The experiments suggest" would be more appropriate.

Line 35, "kinds" should be replaced by "species".

Line 36, "genera" (plural of genus), and the genus names should be capitalized (also lines 37, 38, 206, 213, 254).

Line 42, "similarly" should be deleted here.

Line 43, insert a comma after "receptor-binding".

Line 106, "conserved" instead of "conservative"

Line 125, 20 degrees between what?

Line 524, are shown.

Table S2, what do the authors mean with "artificial residue" in the c footnote?

Fig. 1a, add space between 0.375 and μM.

Fig. 1a and b, the order of the concentration legend could be reversed. Then it would be in the same order as the SRP curves.

Fig. 4a, gB could be labeled as pre- and postfusion.

It is really hard to read Fig. S4. Can it be shown as a 2-page figure or as supplementary data file?

Simon Jenni, Ph.D.
Instructor in Biological Chemistry and Molecular Pharmacology
Harvard Medical School

Response to referees' comments

We appreciate the referees' supportive assessment of our work, and their comments on aspects that could be improved. Below we respond to each referee's points in detail, with notes as to where changes to the manuscript have been made.

Reviewer #1:

The study deals with an interesting new field in the receptor research, the mechanism of host cell entry utilized by various viruses. The experiments in the manuscript are well executed and results properly discussed. Although Eph receptors and ephrins have been, by now, shown to be the entry receptors for several viruses (Henipa viruses, in particular), detailed studies of the entry mechanism are scarce. Thus, this study should be of interest for both Eph receptor investigators and 'functional virologists'.

The study reports crystal structures of Kaposi sarcoma-associated herpesvirus (KSHV) and Epstein-Barr virus attachment proteins in complex with the ligand-binding domain of EphA2. It shows, for the first time, how the viruses utilize a similar binding/recognizing mechanism as the biological EphA2 ligand, ephrin-A1. Interestingly, this mechanism is somewhat similar to the Henipa viruses that use ephrins as entry receptors. The study also demonstrates how the entry mechanism can be manipulated by structure-driven point mutations. Furthermore, the manuscript describes how a specific antibody can inhibit the entry of a virus to epithelial cells.

Thus, the manuscript warrants publication once certain changes have been implemented.

The main issue is language: there are far too many cases to list here but, as an example, a sentence like "Both total of 25 amino acids in LBD were observed…" has to be seriously rewritten and, overall, the whole manuscript has to go through a

serious editing by an independent language reviewer

Response: Thanks for your positive support and kind suggestion. The revised manuscript has been copy-edited for proper English language by services of native English speakers at LetPub.

Other points:

1. Since the Kd of EphA2/LBD binding to EBV/gHgL is 26.3 μM, the authors must emphasize in the methods that the concentration of EphA2/EBV (10 mg/ml) was clearly above this value.

Response: Thanks for your kind suggestion. We repeated the SPR experiments and reanalyzed the data. Please see Page 5, lines 80-82. "The LBD of EphA2 bound to EBV gHgL with approximately 4.12 μM affinity, which was approximately 230-fold lower than the affinity with which the LBD bound to KSHV gHgL (~17.5 nM affinity, Fig. 1a, b)". We also rewrote the methods part, please see Page 18, lines 373-377 in the revised manuscript.

2. Does the difference in the number of glycosylation sites (page 5) in the two gHgL proteins have any biological relevance? If not, omit the sentence on page 5.

Response: Thanks for your kind suggestion. We deleted the sentences in the revised manuscript.

3. "...gL interacts with the 'peripheral region' of LBD..." (page 6) – explain peripheral region in more detail.

Response: Thanks for your kind suggestion. We revised the description. "KSHV gHgL and EBV gHgL mainly bound to two regions of the LBD. In both cases, the N-terminus of gL inserted into the LBD channel, and was shaped like a fishhook. The LBD channel was formed by the D-E, J-K loops and G strand on the two sides, with the base made up of the M stand. In addition, Lloop2 and the β2 sheet of gL interacted with the peripheral region (including the AC loop and D strand) of the LBD channel

like an appressed arm (Fig. 2d, e).” Please see Page 6, lines 117-121 in the revised manuscript.

4. A surface area of 1,180 Å2 is barely ‘larger’ than 994 Å2 (page 7) – don’t use the word ’larger’.

Response: We revised the description. “The buried surface was 1180 $Å^2$ between KSHV gHgL and LBD, slightly larger than that of EBV (993.7 $Å^2$).” According to our structure analysis, we believe that the extensive interactions with more contacts contribute to the higher affinity of KSHV gHgL to LBD than that of EBV gHgL. The result is consistent with the above buried area analysis. Please see Page 7, lines 131-134 in the revised manuscript.

5. ”…the hydrogen bond interaction formed by R103 is crucial…” (page 9) – name the binding partners.

Response: Thanks for your kind suggestion. We revised the description in the revised manuscript on Page 9, lines 176-178. “This reduction was especially noticeable at R103A, indicating that the hydrogen bond interaction formed between R103 and KSHV gL V22 or EBV gL W24 was crucial for the binding of EphA2 to KSHV gHgL or EBV gHgL (Fig. 4b, c).”

6. ”…residues in the core secondary structure of KSHV and EBV gL are more conserved…” – be precise, more than what?

Response: Sorry for the obscure description. We revised this sentence in the revised manuscript on Pages 10-11, lines 214-216. **“**Furthermore, we also found that the residues in the core secondary structures of KSHV and EBV gL were more conserved across the γ-herpesviruses than residues in other positions (Supplementary Fig. 3).”

7. The last paragraph of the results (page 11) – the authors are running through this paragraph; please explain the experiments and the results more elaborately.

Response: Thanks for your kind suggestion. We rewrote this paragraph, please see Pages 11-12, lines 223-239 in the revised manuscript. "We then selected three viruses for further functional validation: Alcelaphine gammaherpesvirus 1 (AIHV-1), Equid gammaherpesvirus 2 (EHV-2), and Murid gammaherpesvirus 4 (MuHV-4): each of these viruses was from one of the other three clusters in the gL phylogenetic tree (Fig. 6b). Plasmids expressing either gH or gL proteins from each of the three viruses were synthesized respectively, and cell-based fusion assays were performed to determine fusion efficiency. As AIHV-1 infects cows, EHV-2 infects horses, and MuHV-4 infects mice, plasmids expressing bovine, equid, and murine EphA2 were also synthesized and evaluated. Cell fusion results showed that the gHgL of HSV-2, which is an α-herpesvirus, could not utilize human EphA2 to trigger cell fusion (Fig. 6d), while KSHV could, indicating that the assay was reliable. Importantly, the gHgL proteins from AIHV-1 and EHV-2 used bovine EphA2 and equid EphA2, respectively, to trigger cell fusion. As the gHgL binding sites for EphA2 were conserved across species (Supplementary Fig. 6b, c), the gHgL proteins of AIHV-1 and EHV-2 could therefore also use human EphA2 to trigger cell fusion (Fig. 6d). Interestingly, MuHV-4 gHgL had the weak ability to use mouse EphA2, but not human EphA2, to trigger cell fusion. Indeed, MuHV-4 may mainly utilize other members of Eph family as the receptors, like RRV. These results suggested that γ-herpesviruses may potentially bind to human EphA2 (or other members of Eph family), highlighting their potential threat to human health."

8. Figure 1c – the figure doesn't show a shift in the elution volume between EBV-gHgL and EBV-gHgL/LBD

Response: Yes, due to the small molecular weight of LBD protein, the peak shift of the complex is not clear. However, the SDS-PAGE of each samples could show whether they could form complex in the gel filtration assays. The SDS-PAGE results in Fig.1c showed that peak 1 contains only EBV gHgL proteins, indicating EBV gHgL and LBD proteins did not form complex in the gel filtration assays. In contrast, the SDS-PAGE results in Fig. 1d showed that peak 1 contains both KSHV gHgL and

LBD proteins, indicating these two proteins could form complex in the gel filtration assays. As expected, EBV gHgL proteins did not form a complex with the LBD protein in the gel filtration assays, while the KSHV gHgL protein did (Fig. 1c, d). We added the description in the revised figure legend of Fig. 1.

Reviewer #2 (Remarks to the Author):

Su et al. present crystal structures of the envelope glycoprotein complexes gHgL from two oncogenic human herpesviruses, Epstein–Barr virus (EBV) and Kaposi's sarcoma-associated herpesvirus (KSHV), in complex with the ligand-binding domain (LBD) of the ephrin receptor tyrosine kinase A2 (EphA2). Both viruses have a tropism, namely, which cell types that they can infect, that is not fully understood. EBV infects human B cells and epithelial cells. The B cell infection pathway is better characterized and a crystal structure of a gHgL/gp42 complex has been reported (see reference 18). Recently, two independent groups identified EphA2 as a possible receptor for EBV during infection of epithelial cells (see references 7 and 8). Su et al. now define the molecular basis for this interaction. They visualize how gHgL of both viruses binds the LBD of EphA2 in a conserved manner. The authors' careful comparison of the binding interfaces provides plausible explanations for their measured
differential affinities of KSHV or EBV gHgL to the host cell receptor domain. As one would predict from their structures, mutations of critical binding interface residues to alanine negatively affects fusion efficiency in a cell-based assay. From the structural interpretation, it is evident that engaging EphA2 by gHgL, a previously reported neutralizing antibody E1D1, or its native ligand is mutually exclusive. The authors conclude their study by showing that envelope glycoproteins from other herpesvirus genera, after comparing their structures to KSHV, are able to promote cell fusion as well when partnered with EphA2.

The manuscript is well presented with well-designed figures that depict the structural results, and an appropriate interpretation of the results. Sufficient details of the

methodology are explained. This study is a significant step forward in our advancement of understanding the pathology of herpesviruses. I do have a few comments.

Major comments:

The English contains many errors and needs editing. Please get help from an editor or native English speaker before resubmitting.

Response: Thanks for your positive support and kind suggestion. The revised manuscript has been copy-edited for proper English language by services of native English speakers at LetPub.

Previously determined crystal structures of gHgL should be referenced in the introduction.

Response: Done. Please see Page 3 lines 46-49 in the revised manuscript. "The crystal structures of gHgL, gp42-HLA-II and gHgL-gp42, in conjunction with the negative-stain electron micrographs of the structure of gHgL-gp42-HLA-II, provided evidence for the dynamic changes leading to the binding of gHgL-gp42 to HLA-II[9-12]."

It is true that on and off rate constants are in principle measured in the surface plasmon resonance (SRP) assays, but the authors only present calculated affinity constants. Instead of writing "quantify the kinetics" (line 76) it would be better to write "measure affinity constants".

Response: Thanks for your kind suggestion. We have modified the sentence in the revised manuscript. Please see Page 5, line 79. "Recent reports demonstrating that EBV and KSHV gHgLs share the same functional receptor EphA2[13,14] prompted us to measure the affinity constants mediating these interactions via surface plasmon resonance (SPR) assays."

The reported unit cell angles for the EBV gHgL-LBD crystal structure are 90.03, 90.167, 89.888 degrees, which is very close to 90 degrees. Can the authors comment on why they assigned space group P1. Does the data not scale in a higher symmetry spacegroup, e.g. it appears that the data can the indexed on a primitive monoclinic or orthorhombic Bravais lattice? How many molecules are in the asymmetric units of the two crystals? Have the authors analyzed the datasets for possible crystal twinning?

Response: We thanks the reviewer for the critical question. The gHgL-LBD diffraction data are once scaled with the orthorhombic space group or monoclinic space group, however the data set could not be decently merged. For example, if we scale it with P2 space group, the R_{merge} for the inner shell (50-5.58 A) is 0.31 (for P1 space group the corresponding R_{merge} is 0.03), indicating that the genuine space group should be P1 instead of any other space group although higher symmetry seems likely plausible.

How well are the N-linked glycans resolved? Did the authors observe electron density for at least the first NAG at each of the positions that is labeled red in Fig. 2b?

Response: Yes, we observed the electron density of NAG at the positions labeled red in the previous Fig. 2b. However, as reviewer 1 suggested, we do not know whether the difference of the number of glycosylation sites in the two gHgL proteins have any biological relevance. Therefore, we omitted the description and graphical representation in the revised manuscript.

In general the figures are excellent and allowed me to understand the science presented very well on their own, something I really appreciate. Ideally, the authors would choose the same color scheme -- at least for the EBV structure -- which was already used for coloring the domains D-I, D-II, D-III and D-IV in the previous paper published in this journal (reference 18). The coloring of the domains in Fig. 2b definitely needs to be changed to make it consistent with the colors used in the other figure panels. For instance, in Fig. 2b domain D-III is yellow, but in Fig. 2a an LBD is yellow, which is confusing. After looking at Fig. S1, I'm not convinced that it is a

good idea to have 2 different coloring schemes, one for Fig. 2a, c-f, and a different one for Fig. 2b and S1. I'm also not sure whether it is necessary to have different colors for the KSHV and EBV structures. The way the authors did it in Fig. S1 (color one in gray if superposed) works very well.

Response: Thanks for your kind suggestion. We regenerated the figures. Please see revised Fig. 2, 3, 5 and Supplementary Fig. 1, 2, 4, 7, 10.

Fig. 2c,f, please define which residues were used for calculating the superposition transformation matrix. Only residues of LBD?

Response: Yes, the superposition is based on residues of LBD. Please see Page 6, lines 114-116 in the revised manuscript. "Interestingly, KSHV gHgL and EBV gHgL were not arranged in a line, but exhibited an angle shift of about 11° relatively to the LBD (Fig. 2c)."

I would replace the right panel of Fig. 2c with Fig. S5b.

Response: Done. Please see revised Fig. 2c.

Simulated annealing omit maps covering the regions shown in Fig. 3e and f should be provided as a supplementary figure. This would allow one to judge how accurate the models might be defined at the binding interface.

Response: Thanks for your kind suggestion. Please see revised Supplementary Fig. 5 for the representative electron density maps for the two complex structures.

In Fig. 3, if I understand correctly, the definition of overlapping residues is not same in Fig. 3c-d (LBD residues that bind gH and gL) and in Fig. 3g (LBD residues that bind KSHV and EBV). This is confusing.

Response: Thanks for your critical question. We checked it again and found they were the same.

Minor comments:

Line 18, please define gHgL in the abstract, e.g. "envelope glycoproteins".

Response: Done. Please see Page 2, lines 17-18 in the revised manuscript. "Viral glycoprotein H (gH) and glycoprotein L (gL) are crucial for the cell tropism by binding to specific receptors."

Line 22, line 64, line 237, the term "network" should not be used here. A network is a system where information is transferred between nodes and therefore an inappropriate term to describe molecular contacts of a protein-protein binding interface.

Response: Thanks for your kind suggestion. We modified the descriptions. Please see lines 22, 67, 251 in the revised manuscript.

Line 25, "proved" is too strong here. "The experiments suggest" would be more appropriate.

Response: Done. Please see line 24 in the revised manuscript.

Line 35, "kinds" should be replaced by "species".

Response: Done. Please see line 33 in the revised manuscript.

Line 36, "genera" (plural of genus), and the genus names should be capitalized (also lines 37, 38, 206, 213, 254).

Response: Done. Please see revised in the paper (Line 34, 35, 36, 212, 219).

Line 42, "similarly" should be deleted here.

Response: Done. Please see line 41 in the revised manuscript.

Line 43, insert a comma after "receptor-binding".

Response: Done. Please see line 42 in the revised manuscript.

Line 106, "conserved" instead of "conservative"

Response: Done. Please see line 109 in the revised manuscript.

Line 125, 20 degrees between what?

Response: Thanks for your kind suggestion. We modified the description. "The Lloop2 of KSHV gL was shorter in length than that of EBV gL, and was shifted about 20° relatively to gL β1 and β2 (Supplementary Fig. 1d, 2a)." Please see lines 103-105 in the revised manuscript.

Line 524, are shown.

Response: Done. Please see line 563 in the revised manuscript.

Table S2, what do the authors mean with "artificial residue" in the c footnote?

Response: This residue is the N-terminal vector-fusion residue. We modified the description as "protein-expression vector-introduced extra residue" in the revised manuscript. We introduced a cleavage site for restriction enzyme between the signal peptide and target gene on the vector to insert the target sequence into the vector conveniently.

Fig. 1a, add space between 0.375 and μM.

Response: Done. Please see revised Fig. 1

Fig. 1a and b, the order of the concentration legend could be reversed. Then it would be in the same order as the SRP curves.

Response: Done. Please see revised Fig. 1

Fig. 4a, gB could be labeled as pre- and postfusion.

Response: Done. Please see revised Fig. 4

It is really hard to read Fig. S4. Can it be shown as a 2-page figure or as supplementary data file?

Response: Done. Please see revised Supplementary Fig. 9.

Reviewer #1 (Remarks to the Author):

The authors have sufficiently addressed my comments and I recommend the manuscript to be accepted.

Reviewer #2 (Remarks to the Author):

The authors have addressed all of the reviewers' comments. After reading the manuscript again, I have no issues with the text and figures of the revised version and recommend publication.

Here are a few remaining typos that can be corrected:

Line 32, Herpesviruses, enveloped double-stranded DNA viruses, …
Line 53, Other members of the EphA family …
Line 114, tip of gH D-I
Line 126, was about 3 Å closer
Line 144, the EBV gL residues
Line 145, the N-terminal region
Line 151, by Van der Waals contacts
Line 177, gHgL. This reduction
Line 198, from α-, β- and γ-herpesviruses
Line 200, α- and β-herpesviruses
Line 224, functional analysis
Line 563 and 566, form a complex
Line 587, Detailed interactions between EBV or KSHV gHgL and EphA2 LBD
Line 602, Schematic diagram of the cell-based fusion assay.
Line 619, inserts into the LBD channel
Line 629, hydrophobic (orange)

Response to referees' comments

We appreciate the referees' supportive assessment of our work, and their comments on aspects that could be improved. Below we respond to each referee's points in detail, with notes as to where changes to the manuscript have been made.

Reviewer #1:

The authors have sufficiently addressed my comments and I recommend the manuscript to be accepted.

Response: Thanks for your positive support.

Reviewer #2:

The authors have addressed all of the reviewers' comments. After reading the manuscript again, I have no issues with the text and figures of the revised version and recommend publication.

Here are a few remaining typos that can be corrected:

Line 32, Herpesviruses, enveloped double-stranded DNA viruses, …

Response: Done.

Line 53, Other members of the EphA family …

Response: Done.

Line 114, tip of gH D-I

Response: Done.

Line 126, was about 3 Å closer

Response: Done.

Line 144, the EBV gL residues

Response: Done.

Line 145, the N-terminal region

Response: Done.

Line 151, by Van der Waals contacts

Response: Done.

Line 177, gHgL. This reduction

Response: Done.

Line 198, from α-, β- and γ-herpesviruses

Response: Done.

Line 200, α- and β-herpesviruses

Response: Done. We added

Line 224, functional analysis

Response: Done.

Line 563 and 566, form a complex

Response: Done.

Line 587, Detailed interactions between EBV or KSHV gHgL and EphA2 LBD

Response: Done.

Line 602, Schematic diagram of the cell-based fusion assay.

Response: Done.

Line 619, inserts into the LBD channel

Response: Done.

Line 629, hydrophobic (orange)

Response: Done.
